# EMT‐cancer cells‐derived exosomal miR‐27b‐3p promotes circulating tumour cells‐mediated metastasis by modulating vascular permeability in colorectal cancer

**DOI:** 10.1002/ctm2.595

**Published:** 2021-12-22

**Authors:** Rongzhang Dou, Keshu Liu, Chaogang Yang, Jinsen Zheng, Dongdong Shi, Xiaobin Lin, Chen Wei, Chunxiao Zhang, Yan Fang, Sihao Huang, Jialin Song, Shuyi Wang, Bin Xiong

**Affiliations:** ^1^ Department of Gastrointestinal Surgery Zhongnan Hospital of Wuhan University Wuhan China; ^2^ Department of Gastric and Colorectal Surgical Oncology Zhongnan Hospital of Wuhan University Wuhan China; ^3^ Hubei Key Laboratory of Tumor Biological Behaviors Wuhan China; ^4^ Hubei Cancer Clinical Study Center Wuhan China; ^5^ Department of Breast Surgery and General Surgery Guangzhou Women and Children's Medical Center Guangzhou China; ^6^ Department of Internal Medicine Affiliated Tumor Hospital of Zhengzhou University Henan Cancer Hospital Zhengzhou China

**Keywords:** colorectal cancer, CTCs, EMT, exosomes, metastasis, miR‐27b‐3p

## Abstract

**Background:**

Metastasis is the main cause of death in colorectal cancer (CRC). Circulating tumour cells (CTCs) are regarded as the precursor cells of metastasis. The CTCs, which underwent epithelial‐mesenchymal transition (EMT), are associated with metastasis and responsible for poor prognosis. EMT cancer cells modulate endothelial permeability in the invasive front and facilitate cancer cell intravasation, resulting in CTCs‐mediated distant metastasis. Exosomes derived from cancer cells are key mediators of cancer‐host intercommunication. However, the mechanism by which EMT‐tumour cells‐derived exosomes modulate vascular permeability and promote CTCs generation has remained unclear.

**Methods:**

Exosomes isolation and purification were conducted by ultra‐centrifugation. Exosomal miRNA was identified by sequencing followed by quantitative PCR. In vitro co‐culture assay experiments were conducted to evaluate the effect of exosomal miR‐27b‐3p on the permeability of blood vessel endothelium. Dual‐luciferase reporter assay, chromatin immunoprecipitation (ChIP) and RNA immunoprecipitation (RIP) were performed to investigate the underlying mechanism by which miR‐27b‐3p is packaged into exosomes. A mouse model was established to determine the role of exosomal miR‐27b‐3p in blood vessel permeability modulation in vivo.

**Results:**

We found that EMT‐CRC cells attenuate the blood vessel barrier by transferring miR‐27b‐3p to human umbilical vein endothelial cells (HUVECs) in exosomes. Mechanically, miR‐27b‐3p atteuated the expression of vascular endothelial cadherin (VE‐Cad) and p120 at the post‐transcriptional level by binding to 3′‐untranslated region of VE‐Cad and p120 directly. The packaging of miR‐27b‐3p into exosomes was induced by heterogeneous nuclear ribonucleoprotein A1 (hnRNPA1), which activated by STAT3. Clinically, miR‐27b‐3p up‐regulated in CRC tissues. Plasma exosomal miR‐27b‐3p was positively correlated with malignant progression and CTC count in CRC patients. Our study reveals a novel mechanism by which EMT‐CRC cells promote metastasis, increasing blood vessel permeability and facilitating the generation of CTCs.

**Conclusion:**

Exosomal miR‐27b‐3p secreted by EMT‐CRC cells increases blood vessel permeability and facilitates the generation of CTCs. Exosomal miR‐27b‐3p may become a promising biomarker for CRC metastasis.

## Graphical abstract

Collectively, our findings revealed that EMT CRC cell‐derived exosomal miR‐27b‐3p transferred to endothelial cells, enhanced blood vessel permeability, and thus facilitated CTC generation by targeting VE‐cadherin and p120. Upstream STAT3 upregulated hnRNPA1 by binding its promotor and hnRNPA1 mediated the packaging of miR‐27b‐3p. Our study highlighted that the STAT3/hnRNPA1/miR‐27b‐3p signal cascade and exosomal miR‐27b‐3p may be promising diagnostic biomarkers for CRC metastasis.

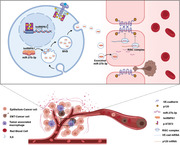

## 1 INTRODUCTION

Metastasis is the main cause of death in colorectal cancer (CRC) patients and progresses in multiple steps.^1^ Circulating tumour cells (CTCs) are considered as the precursor cells of metastasis.^2^ The presence of CTCs, especially CTCs of the epithelial‐mesenchymal transition (EMT) phenotype, is closely related to high metastatic potential in numerous solid cancers, including stomach, hepatocellular and CRCs^3^, ^4^, ^5^ because CTCs that gain mesenchymal traits easily survive and metastasize.^6^ As per a study from our medical center, CRC patients with EMT‐CTCs have worse progression free survival (PFS) and shorter overall survival.^7^ EMT is often activated at the invasive front,^8^ giving the tumour cells their high invasive ability. The activation of EMT is dynamically controlled by various signals from the tumour microenvironment (TME), among which interleukin‐6 (IL6) is one of the most abundant pro‐inflammatory cytokines and promotes EMT by activating snail family transcriptional repressor 1 (SNAIL) and twist family bHLH transcription factor 1 (TWIST1).^9^, ^10^, ^11^ Our group previously revealed that tumour‐associated macrophages (TAMs) secrete IL6 to promote EMT; mesenchymal tumour cells, in turn, enhance the recruitment of TAMs, which form a positive feedback loop and consequently induce liver metastasis.^12^, ^13^ Acquisition of highly invasive traits of tumour cells is the initial step for CTCs‐mediated metastasis. Disrupting through the blood vessel barrier is another crucial step for this process, requiring the disruption of blood vessel tight connection.^14^, ^15^ The disruption of blood vessel integrity and ensuing vascular permeability permit EMT tumour cells' intravasation and subsequent metastasis. However, the mechanisms underlying the entry of EMT tumour cells into the blood vessels by targeting blood vessel integrity and disrupting the blood vessel barrier are not much known. Therefore, exploring these mechanisms by which EMT tumour cells modulate the blood vessel integrity is significant for understanding the EMT‐CTCs mediated metastasis in CRC.

Adherent junctions play a significant role in maintaining rigorous blood vessel integrity by controlling monolayer permeability.^16^ The basal organization of adherent junctions is provided by vascular endothelial cadherin (VE‐Cad), which is enforced by p120‐catenin (p120) and spans the plasma membrane. VE‐Cad binds to VE‐Cad molecules on adjacent endothelial cells through its extracellular domain to form and consolidate the integrity of the endothelium.^17^ Hence, the expression and the regulation of VE‐Cad and p120 are vital for endothelial permeability.^18^ Recent studies have reported that as important carriers, exosomes play a significant role in blood vessel permeability and pre‐metastasis vascular niche formation.^19^, ^20^, ^21^ The mechanisms by which this process occurs are not been entirely elucidated. Investigating how tumour cells‐derived exosomes modulate VE‐Cad and p120 is critical.

Cancer‐derived exosomes contain proteins and RNAs involved in cancer invasion, chemoresistance, immune evasion and metastasis.^22^ Many of these functions have been attributed to exosome‐packaged microRNA (miRNA).^23^ As a small non‐coding RNA, exosomal miRNA can be delivered into the recipient cells and regulate various cellular activities by binding to 3′‐untranslated regions (UTRs) through base pairs.^24^ Furthermore, miRNAs in exosomes are not a simple reflection of the miRNA composition of producer cells, but rather, involved in a highly selective process affected by biogenesis and exosomal contents.^25^, ^26^ RNA binding proteins play an essential role, during this process, binding RNAs and importing RNAs into exosomes. However, the other mechanisms underlying the exosomal packaging in EMT cancer cells and the effects of their exosomal miRNAs on the blood vessel permeability of TME remain poorly defined.

Given the vital position of EMT, blood vessel permeability and CTCs in the process to promote CRC metastasis, we hypothesized that the intercellular communication within EMT cancer cells and blood vessel endothelial cells could mediate CTCs metastasis through increasing vascular permeability. In the present study, we unveiled that miR‐27b‐3p is up‐regulated in CRC tissues, especially in metastatic patients. Besides, miR‐27b‐3p is also enriched in invasive front and associated with EMT. Furthermore, increased plasma exosomal miR‐27b‐3p level was correlated with CTC count in CRC patients. Mechanically, EMT CRC cell‐secreted exosomal miR‐27b‐3p can be transferred into blood vessel endothelial cells and attenuate the VE‐Cad/p120 expression in a post‐transcriptional manner, thereby further inhibiting endothelial junction integrity and promoting cancer metastasis. Moreover, we revealed that the STAT3‐hnRNPA1 pathway participated in the process of miR‐27b‐3p packaging into exosomes, unveiling a novel mechanism of exosomal packaging in EMT cancer cells. These findings indicate a potentially novel intercellular communication mechanism between EMT cancer cells and tumour blood vessels, which promote CRC metastasis by regulating the tumour blood vessel integrity and generation of CTCs.

## 2 RESULTS

### 2.1 EMT tumour cells at invasive front is correlated with lower vascular VE‐Cad and p120 in CRC patients

**FIGURE 1:**
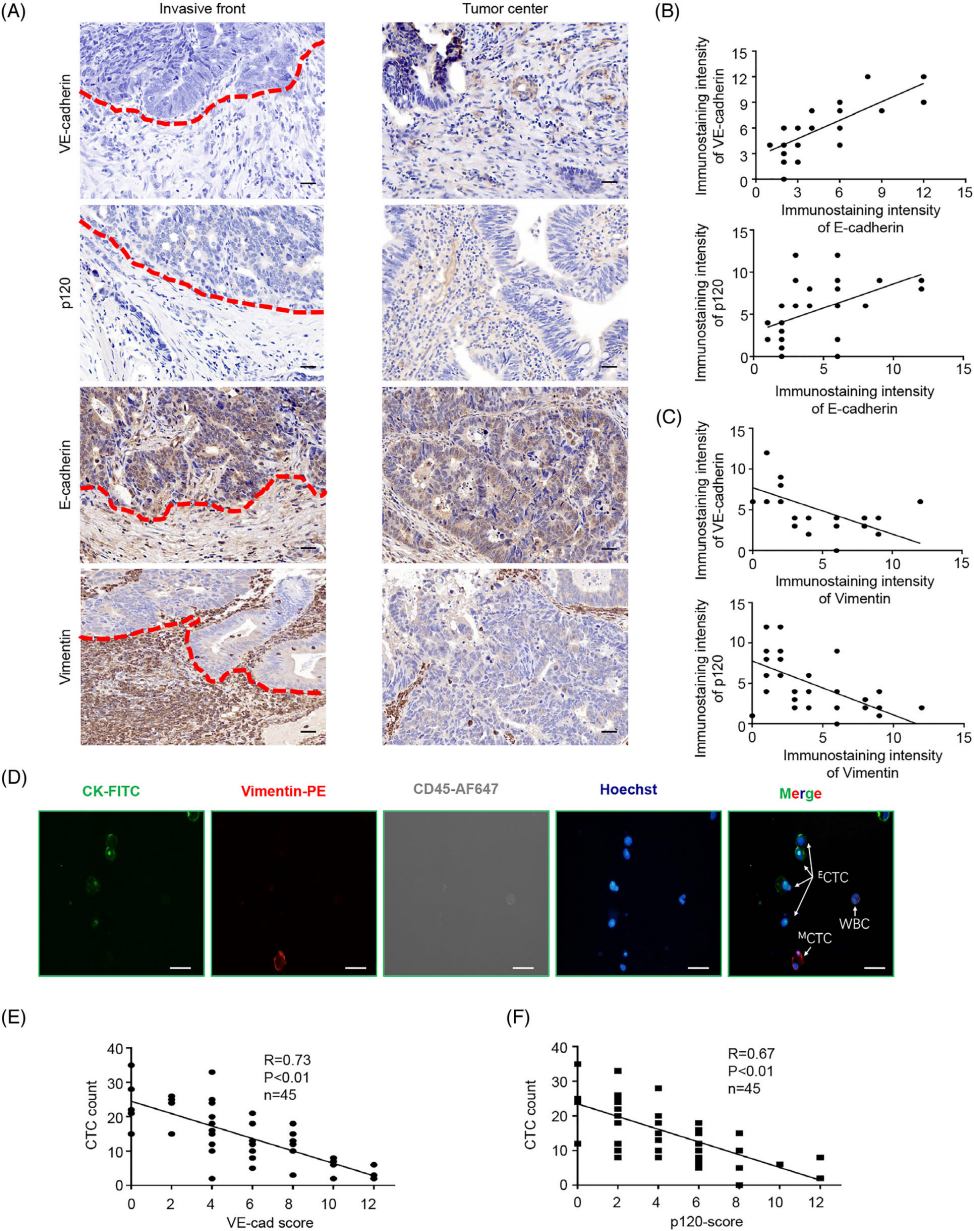
EMT tumour cells at invasive front are correlated with lower vascular endothelial cadherin (VE‐cad) and p120 in colorectal cancer (CRC) patients. (A) Representative immunohistochemistry staining for VE‐cad, p120, and EMT markers in the invasive front and non‐invasive front of serial sections from included patients 3 and 15, respectively. (B and C) Linear regression between immunostaining intensity of VE‐cad/p120 and EMT markers, respectively. (D). Representative circulating tumour cells (CTCs) images. Four‐color immunocytochemistry staining was applied to identify and enumerate CTCs from non‐specially trapped WBCs. Scale bar, 20 μm. (E and F) Association of VE‐cad and p120 expression at invasive front with CTCs count, respectively. Error bars, standard deviation (SD), ns, not significant, *p < .05, **p < .01, ***p < .001

We firstly explored the VE‐Cad/p120 expression and EMT markers in serial sections specimens from 45 CRC patients. We found a low expression of E‐Cad and a conversely robust expression of Vimentin near the invasive front (Figure 1A, Figure S1A,B). Furthermore, a high Vimentin expression was associated with a low expression of VE‐Cad and p120 near the invasive front, indicating higher permeability (Figure 1B,C). Since the tight blood vessel barrier impedes the intravasation of tumour cells, we further explored the relationship between vascular VE‐Cad/p120 and CTC count, CTCs were obtained from 5‐ml peripheral blood of included CRC patients, and CK/CD45 biomarkers were used to identify CTCs and white blood cells (WBCs) (Figure 1D). To our surprise, the expression of vascular VE‐Cad/p120 near the invasive front was negatively correlated with the CTC count (Figure 1E,F). These data indicate that EMT tumour cells associate with blood vessel permeability in TME and imply a possibility that EMT tumour cells promote tumour progression and hematogenous metastasis by regulation of blood vessel permeability.

**HIGHLIGHT**

- Our study illustrated an intercellular communication between mesenchymal cancer cells and blood vessel endothelium.
- IL6, a pro‐inflammatory cytokine, binds to the IL6 receptor (IL6R) that sequentially phosphorylates STAT3 and promotes the EMT program. Otherwise, hnRNPA1 packed miR‐27b‐3p into exosome and delivered into blood vessel endothelium cells.
- miR‐27b‐3p inhibits the level of adherent junction protein VE‐cadherin and p120 thus attenuates blood vessel barrier and facilitates CTCs generation and metastasis.

### 2.2 Exosomal miR‐27b‐3p derived from EMT tumour cells modulates blood vessel barrier

**FIGURE 2:**
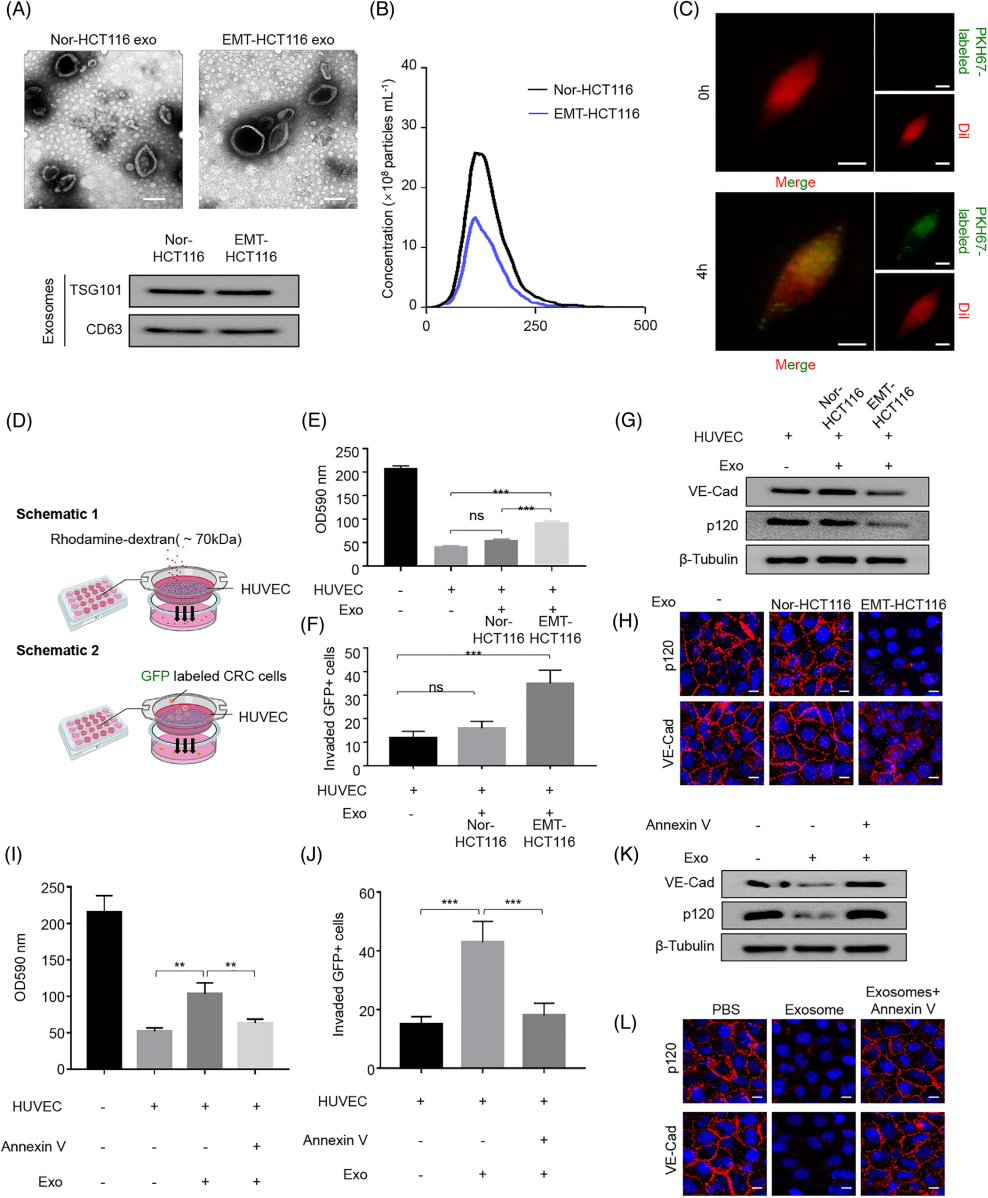
EMT tumour cells‐derived exosomal miR‐27b‐3p modulates blood vessel barrier. (A) Identification of exosomes from HCT116 cells. Upper panel, EM images of secreted exosomes from Nor‐HCT116 and EMT‐HCT116 cells. Scale bar represents 50 nm. Lower panel, the exosomal membrane marker CD63 and TSG101 were analysed by western blot. (B). NanoSight particle‐tracking analysis of size distribution and the number of exosomes from Nor‐HCT116 or EMT‐HCT116. (C). Fluorescent observation in HUVECs after incubation with PKH67‐labelled (green) exosomes derived from EMT‐HCT116 for 0 h and 4 h. Scale bar, 20 μm. (D). Schematic of permeability experiments of HUVEC. Upper panel: Rhodamine‐labelled dextrose molecular probes that can pass through a growing HUVEC monolayer membrane on the filter (.4 μm) were measured for permeability measurement. Lower panel: The number of tumour cells invaded through HUVEC monolayer on the filter (.4 μm) was calculated. (E) HUVEC monolayer membrane pretreated with HCT116/EMT‐HCT116‐derived exosomes. Then add rhodamine–dextran to the upper chamber, and after incubating for 60 min, detect the content of dextran (optical density (OD) 590 nm) in the lower chamber. (F) HUVEC incubated with the exosomes derived from HCT116/EMT HCT116. The number of tumour cells invaded through HUVEC monolayer was calculated. (G) HUVECs were cultured without or with the exosomes derived from HCT116 or EMT‐HCT116 cells. Expression of the vascular endothelial cadherin (VE‐Cad) and p120 proteins was determined by western blot. (H) HUVEC monolayer treated with EMT‐HCT116‐derived exosomes showed a conspicuous diminution of VE‐Cad and p120 expressions compared with Nor‐HCT116. Scale bar, 20 μm. (I) HUVEC incubated with the exosomes derived from EMT HCT116, EMT HCT116+Annexin V. Detect the content of dextran (OD 590 nm) in the lower chamber. (J) HUVEC incubated with the exosomes derived from EMT HCT116, EMT HCT116+Annexin V. Calculate the number of tumour cells invading HUVEC monolayer. (K) HUVECs were cultured without or with the exosomes derived from EMT HCT116, EMT HCT116+Annexin V. Expression of the VE‐Cad and p120 proteins was determined by western blot. (L) Immunofluorescence staining analysis of VE‐cad and p120 expression in HUVECs incubated with EMT‐HCT116 exosomes, EMT‐HCT116 exosomes+ Annexin V. Scale bars represent 20 μm. *p < .05, **p < .01, ***p < .001. exo, exosomes; ns, not significant

The process of EMT acquisition is activated and dynamically controlled by various signals from the TME, among which IL6 is one of the most abundant pro‐inflammatory cytokines in TME. Besides, IL6 is a well‐characterized EMT promoter and works by activating ZEB1, SNAIL and TWIST1.^9^, ^10^, ^11^ Our group previously revealed that TAMs secrete IL6 to promote EMT and consequently induce liver metastasis.^12^ According to the previously described protocols, we selected the CRC cell line with epithelial characteristics (HCT116) and added 50 ng/ml IL6. We found that after IL6 treatment, the expression of EMT‐related protein E‐Cad in CRC cells was reduced, and the expression of Vimentin was up‐regulated (Figure S2A). Besides, we also examined the EMT characteristics (Figure S2B‐D). After acquiring the highly invasive phenotype, traversing through the blood vessel barrier was crucial for the CTCs release and tumour metastasis. We further explored the role of exosomes in intercellular communication between the mesenchymal CRC cells and the blood vessel endothelium. Exosomes isolated from cell culture media of control cells and EMT‐HCT116 cells had the typical disc‐shaped morphology and around 100 nm in diameter. Exosomal membrane markers TSG101 and CD63 were detected by western blot to confirm the isolation of exosomes (Figure 2A). We further conducted NanoSight analysis to the collected product which exhibited median diameter of 130.3 nm and 127.0 nm, respectively (EMT‐HCT116 and Nor‐HCT116) (Figure 2B). Next, the PKH67‐labelled exosomes from EMT‐HCT116 were further used to treat human umbilical vein endothelial cells (HUVECs). Green PKH67 was observed in recipient HUVECs after incubation for 4 h (Figure 2C). These findings suggest that EMT‐HCT116‐derived exosomes can be delivered to HUVECs. On this basis, we further treated HUVECs with exosomes purified from Nor/EMT HCT116 cells. Permeability and tube formation assays were performed in vitro to detect whether exosomes secreted by EMT CRC cells regulate vascular permeability and angiogenesis. Rhodamine‐labelled dextrose molecular probes were measured for permeability measurement as reported by Zhou et al.^20^ (Figure 2D). Compared with the exosomes from control cells, exosomes from EMT‐HCT116 cells obviously promoted vascular permeability (Figure 2E). The detection of green fluorescent protein (GFP)‐labelled tumour cells that penetrated the HUVEC monolayer in transendothelial cell invasion assay confirmed these findings (Figure 2F, Figure S3A). However, exosomes derived from EMT‐HCT116 cells had no enhancement effect on HUVEC tube formation compared with HCT116 cells (Figure S3B). Western blot and immunofluorescence assays indicated that VE‐Cad and p120 rather than ZO‐1 or Occludin level in HUVEC decreased dramatically in EMT‐HCT116 exosome treated group (Figure 2G,H, Figure S3C). After treatment with an exosome internalization inhibitor, Annexin V,^27^ Exosomes from EMT‐HCT116 failed to induce the increased permeability in HUVECs (Figure 2I,J, Figure S3D), and western blot and immunofluorescence indicated the expression of VE‐Cad/p120 was restored (Figure 2K,L). Taken together, our results indicate that EMT‐HCT116‐secreted exosomes can manipulate HUVECs permeability through exosomes.

### 2.3 EMT tumour cells secreted exosomal miR‐27b‐3p can be transported into vascular endothelial cells

**FIGURE 3:**
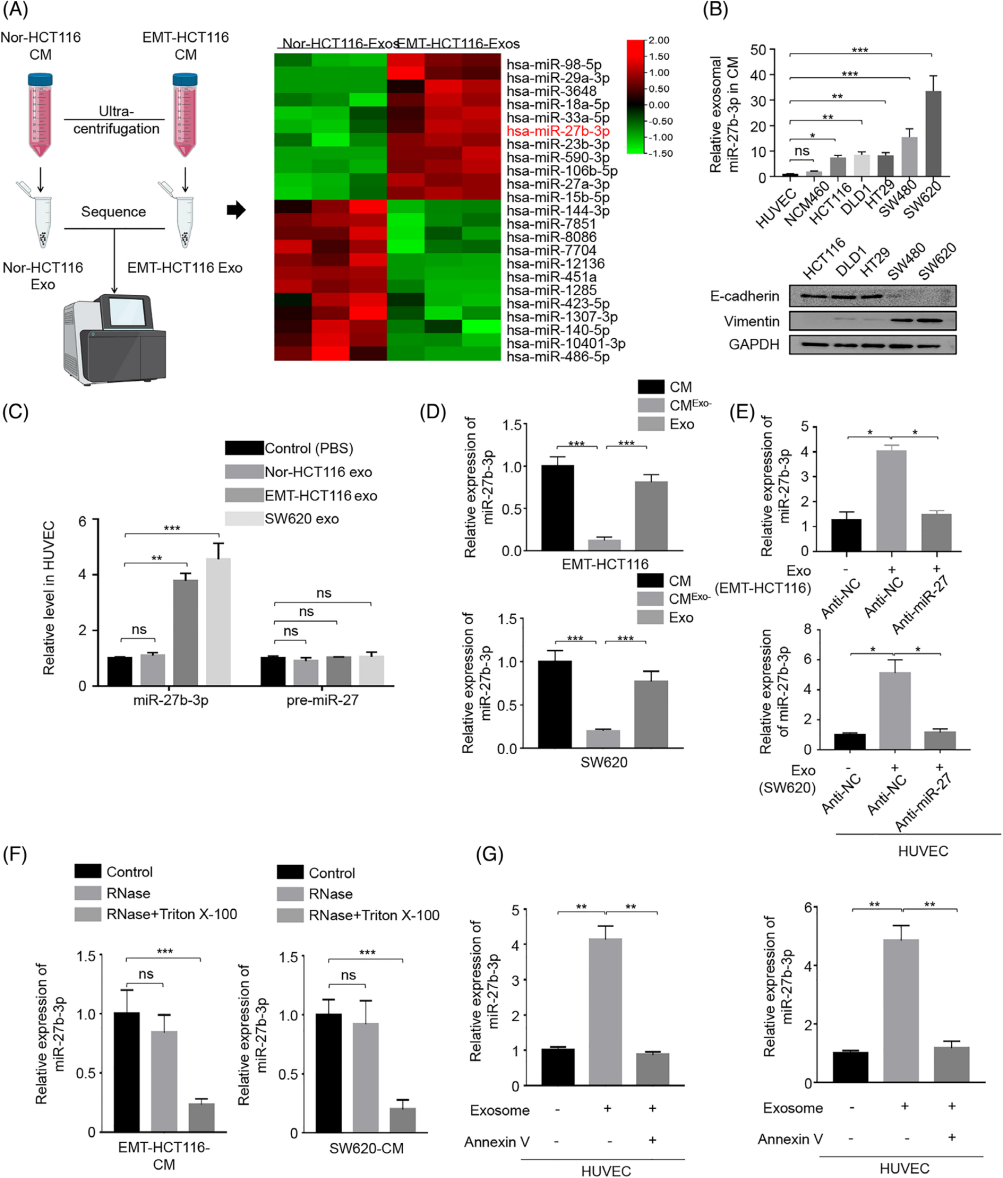
The exosomal miR‐27b‐3p secreted by colorectal cancer cells is transmitted into endothelial cells. (A) Heatmap showing the overall expression change of miRNAs in exosomes secreted by Nor‐HCT116 and EMT‐HCT116 cells. (B) The level of miR‐27b‐3p within HUVECs and the indicated colorectal cell lines in their corresponding conditioned media (CM) was analysed by qPCR. cel‐miR‐67 as an external control for secreted miR‐27b‐3p in CM. Western blotting was used to reveal the expression of EMT phenotype in colorectal cancer cell lines with E‐cadherin and Vimentin. (C) The level of miR‐27b‐3p or pre‐miR‐27b‐3p in HUVEC incubated with exosomes from different cells was analysed by RT‐PCR. (D) RT‐PCR analysis of miR‐27b‐3p in the CM, exosome‐depleted CM, and exosome derived from EMT‐HCT116 cells and SW620. (E). Anti‐endogenous miR‐27b‐3p in EMT‐HCT116 cells/SW620 attenuates the ability of EMT‐HCT116/SW620‐derived exosomes to enhance miR‐27b‐3p levels in the receptor HUVEC. HUVEC was cultured with or without exosomes from EMT‐HCT116/SW620 cells transfected with NC/miR‐27b‐3p‐inhibitor. (F). RT‐PCR analysis of miR‐27b‐3p in the CM of EMT‐HCT116/SW620 cells treated with RNase (2 mg/ml) alone or with Triton X‐100 (.1%) on this basis. (G) HUVEC treated with or without Annexin V was incubated with the exosomes derived from EMT HCT116/SW620. miR‐27b‐3p level was measured by RT‐PCR. *p < .05, **p < .01, ***p < .001. exo, exosomes; NC, negative control; ns, not significant

In the light of the significance of exosomal miRNAs in intercellular communication, we sequenced Nor‐HCT116‐exosomes and EMT‐HCT116‐exosomes by Illumina sequencing (SRA database: SRP227899) (Figure 3A). Based on various database predictions (miRWalk, Starbase, miRmap), miR‐27b‐3p was the most up‐regulated miRNA in the EMT‐HCT116 exosomes which increased HUVECs permeability (Figure S4A‐C). On this basis, the expression levels of the miR‐27b‐3p in human CRC cell lines (DLD‐1, SW620, HT29, HCT‐116, SW480) and NCM460 were measured by quantitative reverse transcriptase PCR (qRT‐PCR). Significantly increased levels of the secreted‐miR‐27b‐3p were detected in two distinct mesenchymal CRC cell lines (SW620 and SW480),^28^, ^29^ while HUVEC and NCM460 cells expressed low levels of miR‐27b‐3p (Figure 3B). We then investigated whether CRC cells can secrete exosomal miR‐27b‐3p and transfer them into endothelial cells. We found that the pre‐miR‐27b‐3p level did not change significantly in the HUVEC receptor (Figure 3C). This finding indicated that the incubation of exosomes derived from EMT‐CRC cells did not alter endogenous miR‐27b‐3p biosynthesis in HUVECs. After extracting CRC cell exosomes, we found that miR‐27b‐3p levels in exosomes and the entire CRC‐conditioned medium (CM) were nearly equal (Figure 3D). After incubating HUVEC with exosomes/CM from miR‐27b‐3p inhibitor‐transfected EMT‐HCT116 cells and SW620, we observed a significant decrease of miR‐27b‐3p in the recipient HUVEC cell (Figure 3E; Figure S5A). We then observed that transfected miR‐27b‐3p inhibitor in SW620 decreased the miR‐27b‐3p level in CM and exosomes (Figure S5B). Moreover, the extracellular level of miR‐27b‐3p in CRC‐CM was not affected by RNase A, and after the simultaneous treatment of RNase A and Triton X‐100 (Figure 3F), the level of miR‐27b‐3p in CRC‐CM was markedly reduced. These observations suggest that the extracellular miR‐27b‐3p is not directly released but packaged in the membrane and transported. Besides, the miR‐27b‐3p level in annexin V‐treated HUVECs decreased after incubation with EMT‐HCT116/SW620 exosomes (Figure 3G). These observations indicate that CRC cells secrete exosomal miR‐27b‐3p that is transported into the vascular endothelial cells.

### 2.4 Exosomal miR‐27b‐3p disrupts the connection between vascular endothelial cells by targeting p120 and VE‐Cad in a post‐transcriptional manner

**FIGURE 4:**
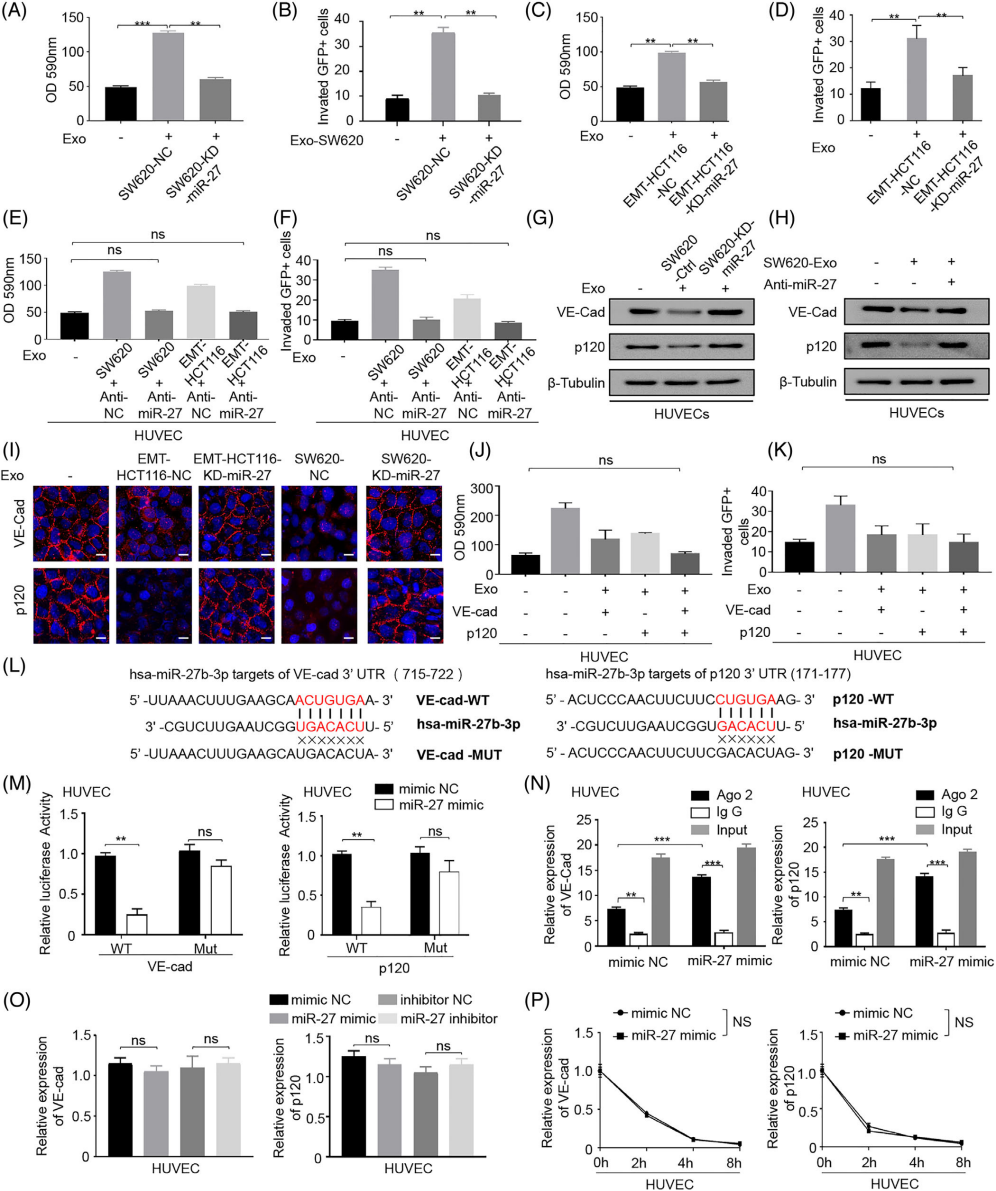
The exosomal miR‐27b‐3p disrupts the connection between vascular endothelial cells by targeting p120 and vascular endothelial cadherin (VE‐Cad) in a post‐transcriptional manner. (A). Transfect HUVEC with the exosomes derived from SW620‐KD‐miR‐27b‐3p or their control sublines (SW620‐NC). Then add rhodamine–dextran to the upper chamber, and after incubating for 60 min, detect the content of dextran (OD 590 nm) in the lower chamber. (B) Transfect HUVEC with the exosomes derived from SW620‐KD‐miR‐27b‐3p or their control sublines (SW620‐NC). It was found that the permeability of the exosomes‐treated group of SW620‐miR‐27b‐3p increased significantly. Calculate the number of tumour cells invading HUVEC monolayer. (C) Transfect HUVEC with the exosomes derived from EMT‐HCT116‐KD‐miR‐27b‐3p or their control sublines (EMT‐HCT116‐NC). Then add rhodamine–dextran to the upper chamber, and after incubating for 60 min, detect the content of dextran (OD 590 nm) in the lower chamber. (D) Transfect HUVEC with the exosomes derived from EMT‐HCT116‐KD‐miR‐27b‐3p or their control sublines (EMT‐HCT116‐NC). It was found that the permeability of the exosomes‐treated group of EMT‐HCT116‐KD‐miR‐27b‐3p increased significantly. Calculate the number of tumour cells invading HUVEC monolayer. (E) Transfect HUVEC with anti‐NC or miR‐27b‐3b inhibitor for 1 day, inoculate it on the filter membrane of Transwell chamber, and then incubate with SW620 or EMT‐HCT116‐derived exosomes for 2 days. Then add rhodamine–dextran to the upper chamber, and after incubating for 60 min, detect the content of dextran (OD 590 nm) in the lower chamber. (F) HUVEC was transfected with anti‐NC or miR‐27b‐3b inhibitor for 1 day, inoculated on the filter membrane of Transwell chamber, and incubated with SW620 or EMT‐HCT116‐derived exosomes for 2 days in the next step. Calculate the number of tumour cells invading HUVEC monolayer. (G) The exosomes secreted by miR‐27‐knock down SW620 cells were incubated with HUVEC. Expression of the VE‐Cad and p120 proteins was determined by western blot. (H) The exosomes secreted by SW620 cells were incubated with HUVEC. On this basis, antagonize endogenous miR‐27 in SW620 cells. Expression of the VE‐Cad and p120 proteins was determined by western blot. (I) Immunofluorescence staining analysis of VE‐cad, p120 expression in HUVECs incubated with Nor‐HCT116/ EMT‐HCT116 or SW620‐KD‐ miR‐27/SW620NC or derived exosomes. Scale bar, 25 μm. (J) HUVECs were incubated with exosomes from SW620 with or without VE‐cad/p120 co‐expression, then add rhodamine–dextran to the upper chamber, and after 60 min, detect the content of dextran (OD 590 nm) in the lower chamber. (K) HUVECs were incubated with exosomes from SW620 with or without VE‐cad/p120 co‐expression, calculating the number of tumour cells invading HUVEC monolayer. (L) miR‐27b‐3p inhibits the luciferase activity controlled by wild‐type VE‐Cad and p120 3ʹ‐untranslated region (UTR) but does not affect the luciferase activity controlled by mutant VE‐Cad and p120 3ʹUTR. A schematic representation of the pmiR‐RB‐REPORTTM dual‐luciferase reporter vector with 3′‐UTR of P120/VE‐Cad mRNA harbours miR‐27b‐3p cognate sites. Assays were performed in triplicates. (M) HUVEC cells were transfected with miR‐27b‐3p mimic or mimic negative control (mimic NC), and the relative luciferase activity of the reporter plasmid carrying wild‐type, mutant P120/VE‐Cad 3′‐UTR was further detected. (N) AGO2‐RNA immunoprecipitation experiments (RIP) showed that miR‐27b‐3p interacts with P120/VE‐Cad in HUVEC cells. (O) HUVEC cells transfected with mimic NC/miR‐27 mimic/inhibitor NC/miR‐27 inhibitor. Quantitative reverse transcriptase PCR (qRT‐PCR) was used to detect P120/VE‐Cad mRNA levels. (P) Stability of the VE‐cad/p120 after miR‐27b‐3p overexpression. Levels of VE‐cad/p120 mRNA in HUVECs were examined at different times after administration with actinomycin D. Error bars, SD, *p < .05, **p < .01, ***p < .001. exo, exosomes; NC, negative control; ns, not significant

We further explored the role of exosomal miR‐27b‐3p in tumour cell transendothelial invasion. Penetration of rhodamine‐labelled dextran in HUVEC monolayer co‐cultured with exosomes was measured to determine the endothelial cell permeability in vitro. We generated SW620‐KD‐miR‐27b‐3p using lentiviral transfection. The transfection efficiency was evaluated with fluorescence and qRT‐PCR (Figure S6A). SW620 cell‐derived exosomes increased the ability of transendothelial invasion of cancer cells, and SW620‐KD‐miR‐27b‐3p cell‐derived exosomes inhibited transendothelial invasion (Figure 4A). The detection of GFP‐labelled tumour cells that penetrated the HUVEC monolayer in a transendothelial cell invasion assay also confirmed the above results (Figure 4B, Figure S7A). In addition, exosomes secreted by EMT‐HCT116 cells promoted rhodamine penetration. EMT‐HCT116‐KD‐miR‐27 cell‐derived exosomes inhibited transendothelial invasion (Figure 4C,D, Figure S7B). The penetration of rhodamine and trans‐endothelial invasion decreased in HUVEC transfected with miR‐27b‐3p inhibitor (Figure 4E,F, Figure S7C). Western blot and immunofluorescence assays showed that exosomes derived from EMT‐HCT116/SW620 reduce the VE‐Cad/p120 protein levels in HUVECs, and knockdown miR‐27b‐3p restored the expression of VE‐Cad/p120 (Figure 4G–I). In the rescue experiment, HUVECs were treated with the exosomes secreted by SW620 cells; the permeability of HUVEC was restored after VE‐Cad/p120 was up‐regulated (Figure 4J,K, Figure S7D,E). Finally, we performed a dual‐luciferase reporter gene experiment by cloning VE‐Cad/p120 3′‐UTR into the luciferase reporter plasmid. Both VE‐Cad and P120 contained a conserved miR‐27b‐3p homology site, 715–722 for VE‐Cad 3‐UTR and 171–177 for p120 3′‐UTR, respectively (Figure 4L), which were predicted target sites of miR‐27b‐3p. Moreover, the miR‐27b‐3p mimic or mimic negative control (NC) was co‐transfected using a luciferase reporter vector pmiR‐RB‐REPORTTM‐VE‐Cad/ P120 3′‐UTR and a mutation reporter vector, respectively, which carried an miR‐27b‐3p binding site mutation. miR‐27b‐3p inhibited the luciferase activity of wild‐type VE‐Cad / p120 3′‐UTR‐transfected HUVEC cells without affecting the mutant VE‐Cad/p120 3′ ‐UTR carrier cell luciferase activity (Figure 4M). miRNA is reported to affect target protein levels through affecting the mRNA stability or inhibiting mRNA translation by forming the RNA‐induced silencing complex complex.^30^ The AGO2 protein and RNA immunoprecipitation test (RIP) was performed, in which no statistical dissimilarity in the expression level of AGO2 protein between miR‐27b‐3p mimic and mimic NC groups was found. Compared with IgG immunoprecipitation, AGO2 immunoprecipitation revealed enhanced VE‐Cad/p120 mRNA expression (Figure 4N). To explore the specific mechanism of miR‐27b‐3p regulating VE‐Cad/p120, we transfected HUVECs with miR‐27b‐3p mimic, inhibitor and corresponding NC and detected the expression of p120/VE‐Cad mRNA by qRT‐PCR. No difference was found in VE‐Cad/p120 mRNA expression across different groups (Figure 4O). miR‐27b‐3p mimic or corresponding mimic NC was applied to transfect HUVECs, after 24 h actinomycin D was administrated and VE‐Cad/p120 mRNA levels were measured at specified time points (Figure 4P). On this basis, we characterized polysome‐associated VE‐Cad/p120 mRNA in miR‐27b‐3p manipulated HUVECs. co‐location These findings suggested that miR‐27b‐3p inhibits VE‐Cad/P120 protein at the post‐transcriptional level by binding to 3′‐UTR of VE‐Cad and p120 directly, destroying the integrity of the endothelial connection and increasing vascular permeability.

### 2.5 STAT3‐up‐regulated hnRNPA1 mediated the packaging of miR‐27b‐3p into exosomes

**FIGURE 5:**
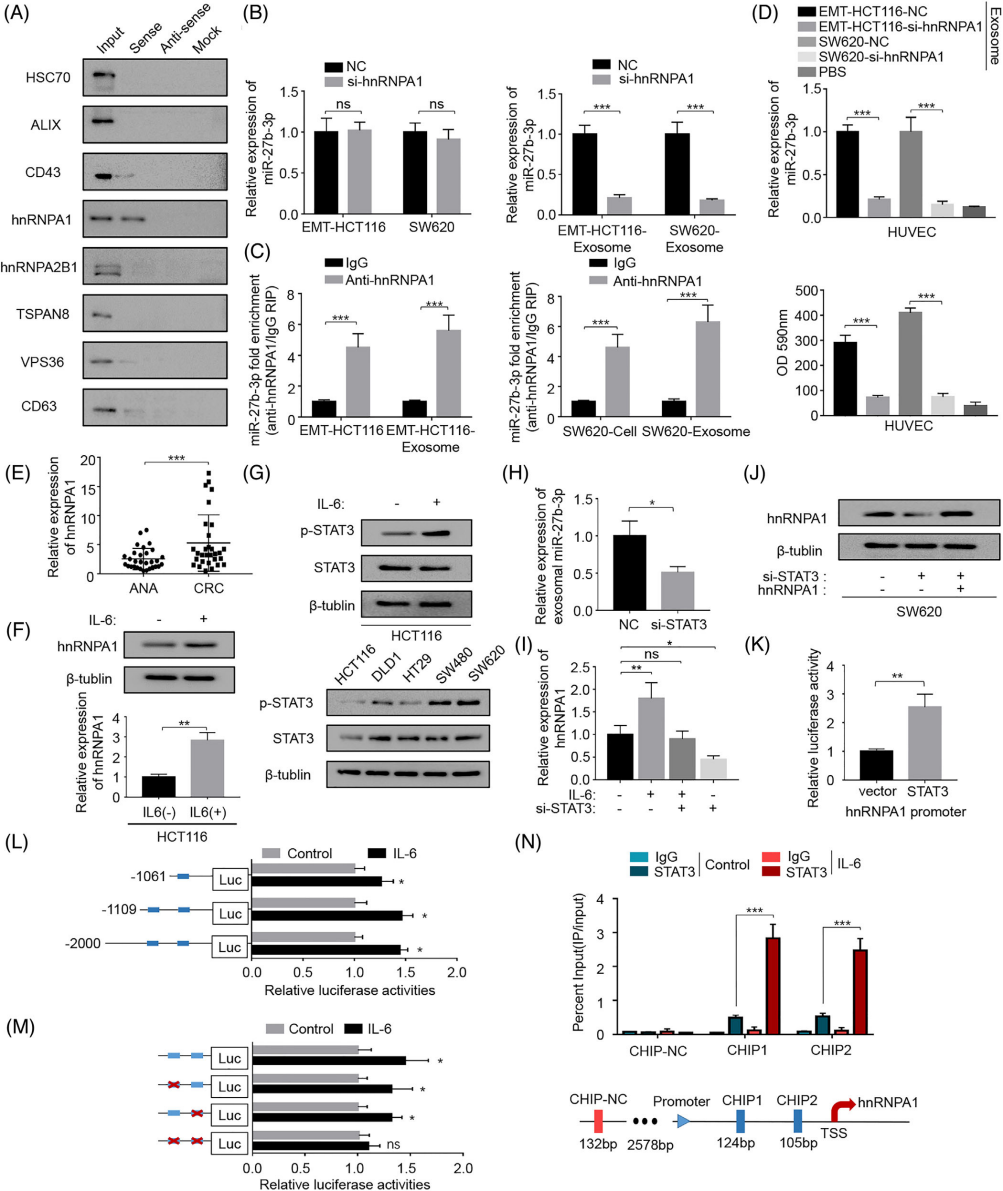
STAT3‐up‐regulated hnRNPA1 mediated the packaging of miR‐27b‐3p into exosomes. (A) Western blot analysis of indicated proteins in precipitates retrieved by in vitro transcribed biotin‐labelled miR‐27b or antisense RNA in the cytoplasmic lysates of HCT116 (mesenchymal type) cells. (B) qRT‐PCR analysis of miR‐27b‐3p in colorectal cancer (CRC) cells (EMT‐HCT116 and SW620) and exosomes after silence hnRNPA1. (C) RNA immunoprecipitation (RIP) assay to determine the enrichment of hnRNPA1 on miR‐27b‐3p relative to IgG in cytoplasmic, or exosomal lysates of EMT‐HCT116 and SW620 cells. (D) Upper panel: HUVECs were incubated with exosomes from cells as indicated, the miR‐27b‐3p in recipient HUVECs was determined by qRT‐PCR. Lower panel: HUVECs were incubated with exosomes from cells as indicated, then add rhodamine–dextran to the upper chamber, and after 60 min, detect the content of dextran (OD 590 nm) in the lower chamber. (E) The relative expression level of hnRNPA1 in CRC tissues and paired normal tissue(*n* = 30). (F) Expression of hnRNPA1 after treatment HCT116 cells with IL6 (50 ng/ml) or control for 48 h. (G) Upper panel: Expression of STAT3 and p‐STAT3 after treatment HCT116 cells with IL6 (50 ng/ml) or control for 48 h. Lower panel: Level of p‐STAT3/STAT3 was determined through western blot in indicated cell lines. (H) qRT‐PCR results of exosomal miR‐27b‐3p derived from SW620 normalized as to expression of U6; Error bars, SD. (I) The hnRNPA1 protein expression was detected by western blot after transfection with si‐STAT3 alone or in combination with hnRNPA1. (J) qRT‐PCR was conducted to testify the hnRNPA1 level in HCT116 cells transfected with STAT3 siRNAs (si‐STAT3) and incubated with IL6 for 48 h afterwards. (K) Luciferase reporter assays were carried out using HCT116 cells overexpressing STAT3 or the vector control. (L) Serially truncated and mutated hnRNPA1 promoter constructs were cloned into pGL3‐basic luciferase reporters, which transfected to HCT116 cells to identify STAT3 binding regions in the hnRNPA1 promoter. The relative luciferase activities were determined after IL6 (50 ng/ml) treatment for 4 h. Selective mutation analyses identified STAT3‐responsive regions in the hnRNPA1 promoter in HCT116 cells; Error bars, SD. (M) Schematic presentation of the chromatin immunoprecipitation (CHIP) localization of the hnRNPA1 promoter region. Chromatin immunoprecipitation (ChIP) assay demonstrated STAT3 directly to the hnRNPA1 promoter, including non‐specific control (NC), CHIP1, and CHIP2 in HCT116 cells cultured in the absence (blue) or presence (red) of IL6. Chromatin of HCT116 was subjected to immunoprecipitation with STAT3 antibodies; IgG was used as negative control. Input, 2% of total lysate. Error bars, SD, *p < .05, **p < .01, ***p < .001. exo, exosomes; NC, negative control; ns, not significant

Since the expression pattern in primary CRC cell lines did not conform with the exosomal miR‐27b‐3p level, indicating the cellular miR‐27b‐3p level is not the decisive factor of exosomal miR‐27b‐3p, we next delved into the specific packaging pattern of extracellular miR‐27b‐3p during the EMT process (Figure S8A,B). We performed RNA pulldown experiments on cytoplasmic extracts and found that the miR‐27b‐3p probe binds explicitly to heterogeneous ribonucleoprotein A1 (hnRNPA1) among several proteins reportedly involved in the process of exosomes packaging^31^ (Figure 5A). We specifically knocked down hnRNPA1 using small interfering RNA (siRNAs) and found that the expression level of miR‐27b‐3p in CRC cells was almost unchanged. By contrast, hnRNPA1 knockdown completely restored the miR‐27b‐3p level in exosomes after IL6 treatment (Figure 5B, Figure S8C,D). Accordingly, we speculated that hnRNPA1 is involved in miR‐27b‐3p package in exosomes. To further validate the binding of miR‐27b‐3p on hnRNPA1, we performed RIP experiments in CRC cells and found a higher miR‐27b‐3p content in the hnRNPA1 antibody group than the IgG group (Figure 5C). To further confirm the role of hnRNPA1 in the package of miR‐27b‐3p, expression level of miR‐27b‐3p in recipient HUVECs treated with exosomes from si‐hnRNPA1‐transfected EMT‐HCT116 cells and SW620 was evaluated, and we observed a decrease of miR‐27b‐3p in the recipient HUVEC. Besides, permeability assay indicated permeability of HUVECs was restored by hnRNPA1 knockdown (Figure 5D). hnRNPA1 is an RNA‐binding protein that mediates encapsulation of multiple miRNAs into exosomes.^24^, ^32^ We observed a significant increase of hnRNPA1 in CRC tissues by qRT‐PCR(Figure 5E, Figure S9A). Moreover, the co‐location of hnRNPA1 and miR‐27b‐3p was also verified in immunofluorescence confocal microscopy (Figure S9B). We next explored whether hnRNPA1 is the cause of the increased EMT CRC cells exosomal miR‐27b‐3p. Firstly, we observed the protein and mRNA level of hnRNPA1 increased in EMT‐HCT116 (Figure 5F). Yao et al. recently reported that STAT3 can transcriptionally increase the level of hnRNPA1.^33^ Considering the aberrant expression and activation of STAT3 in CRC during the EMT process, we hypothesized that STAT3 promotes miR‐27b‐3p packaging into the exosomes by increasing the level of hnRNPA1. Next, we detected STAT3 and phosphorylated STAT3 levels. The phosphorylated levels in HCT116 cells that underwent EMT were up‐regulated, and STAT3 was highly activated in two mesenchymal CRC cell lines as well (SW480, SW620) (Figure 5G). Then, we assessed the exosomal miR‐27b‐3p in SW620 after knocking down STAT3, qRT‐PCR was conducted, and results showed a significant decrease in the exosomal miR‐27b‐3p level after STAT3 was knocked down (Figure 5H). To further confirm the up‐regulation effect of IL6‐STAT3 on hnRNPA1, we evaluated the level of hnRNPA1 in HCT116 cells. We observed that the up‐regulation of hnRNPA1 after IL6 treatment was significantly attenuated after STAT3 was knocked down by siRNAs (Figure 5I). Similar results were obtained after treatment with Stattic, an STAT3 inhibitor (Figure S9C). The rescue experiments with si‐STAT3 and hnRNPA1 overexpression vector co‐transfected into SW620 cells confirmed the above findings (Figure 5J). To further explore the underlying mechanism by which STAT3 up‐regulated hnRNPA1 levels, we cloned the hnRNPA1 promoter into the Luc reporter construct. The overexpression of STAT3 significantly increased the promoter activity of hnRNPA1 (Figure 5K). Furthermore, to determine whether STAT3 directly activated the transcription of hnRNPA1, we conducted bioinformatics analysis (with the JASPAR database, threshold 90%) to screen for potential binding sites located within 2 kb upstream region of the hnRNPA1 transcription start site. There are two potential binding sites located in this region (Figure S9D). STAT3‐induced promoter activity was significantly decreased in hnRNPA1 promoter truncation mutants treated with IL6 (Figure 5L). The site‐directed mutation of both of these potential binding sites significantly reduced the STAT3‐induced activation of the promoter (Figure 5M). Furthermore, we designed primer sets in the chromatin immunoprecipitation (ChIP) assay to specifically amplify the part of hnRNPA1 promoter regions that contained two putative STAT3 binding sites. The strong binding of STAT3 on the promoter region of hnRNPA1 was confirmed, and the binding increased in the presence of IL6 (Figure 5N). Collectively, these results indicated that in EMT CRC cells, activated STAT3 transcriptionally up‐regulated hnRNPA1, mediating the enhanced secretion of exosomal miR‐27b‐3p.

### 2.6 Colorectal cancer cell‐secreted miR‐27b‐3p enhanced tumour metastasis in vivo

**FIGURE 6:**
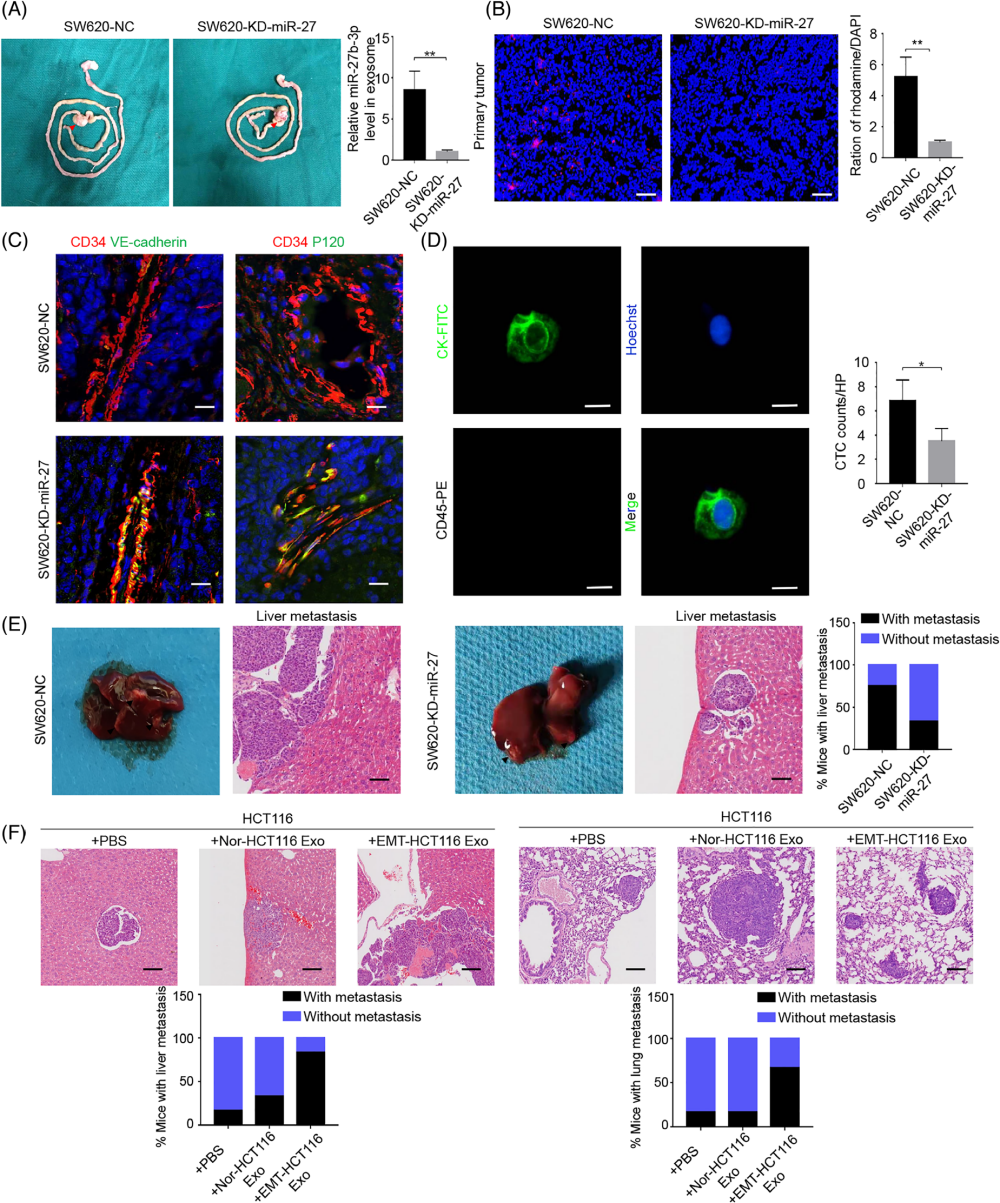
Colorectal cancer cell‐secreted miR‐27b‐3p enhanced tumour metastasis in vivo. (A) miR‐27b‐3p levels in the exosomes from plasma of mice bearing orthotopic implantation with SW620‐KD‐miR‐27b‐3p (*n* = 6) or its control lines (*n* = 8) increased. Error bars, SD. The miR‐27b‐3p levels were measured by qRT‐PCR and normalized to cel‐miR‐67 as external control. (B) The vascular permeability of primary tumour increased. Orthotopic tumour‐bearing mice with SW620‐KD‐miR‐27b‐3p (*n* = 6) or its control lines (*n* = 8) were intravenous injected with rhodamine. Levels of rhodamine–dextran with red fluorescence in frozen tissues section under fluorescent microscopy were normalized to the levels of 4',6‐diamidino‐2‐phenylindole. Error bars, SD. Scale bar, 100 μm. (C) Representative fluorescence in situ hybridization/ immunofluorescence (FISH/IF) staining for vascular endothelial cadherin (VE‐Cad) (green) /P120 (green) and CD34 (red). The vascular structures were labelled by CD34 (red). Scale bar represents 100 μm. (D). The circulating tumour cells (CTCs) counts decreased in mice bearing SW620‐KD‐miR‐27 (*n* = 6); representative CTCs images from mice bearing SW620‐miR‐27b‐3p were shown. Scale bar, 20 μm. (E) Percentage of mice with metastasis is indicated from SW620‐NC/SW620‐KD‐miR‐27b‐3p. Representative images of metastatic lesions in the liver from mice in the SW620‐NC/SW620‐KD‐miR‐27b‐3p. Scale bar represents 100 μm. (F) Upper panel: Representative images of metastatic lesions in the liver and lung from mice in the Nor‐HCT‐116‐bear mice that received indicated treatment. Scale bar represents 100 μm. Lower panel: Percentage of mice with liver and lung metastasis is indicated. Error bars, SD, *p < .05, **p < .01, ***p < .001

To determine the role of exosomal miR‐27b‐3p on the disruption of the blood vessel barriers and induction of in vivo cancer metastasis, SW620‐KD‐miR‐27b‐3p and control cell lines were implanted into the end of the murine caecum. The circulating exosomal miR‐27b‐3p level was measured by qRT‐PCR. And, the exosomal miR‐27b‐3p level in plasma derived from orthotopic bearing mice with SW620‐KD‐miR‐27b‐3p was lower than the control cell xenograft‐bearing mice; furthermore the tumour weight and tumour volume of SW620‐KD‐miR‐27b‐3p cell line group were significantly decreased when compared with control cell line group (Figure 6A, Figure S10A). To confirm the in vivo effect of vascular permeability, we injected fluorescent rhodamine–dextran into the tail vein of orthotopic‐bearing mice. Much more rhodamine–dextran was extravasated into xenografts in SW620‐Ctrl‐bearing mice under a fluorescent microscope than SW620‐KD‐miR‐27b‐3p (Figure 6B), indicating a dramatically higher vascular permeability at the primary tumour site. Compared with the SW620‐NC cell line, the SW620‐KD‐miR‐27b‐3p cell line group had higher expression of VE‐Cad and p120 in tumour blood vascular endothelial cells (Figure 6C). Since the leakage between endothelial cells facilitated extravasion of the tumour cells into the blood vessels, we next examined CTCs with blood film and CTCs biopsy technology in mice. Isolated CTCs were identified with specific immunofluorescent markers, and a lower CTCs count was detected in SW620‐KD‐miR‐27b‐3p‐bearing mice (Figure 6D). In addition, tumour metastasis status was quantified in the serial sections of the livers and lungs. More distant metastasis was observed in SW620‐NC‐bearing mice than the mice of the SW620‐KD‐miR‐27b‐3p group (Figure 6E, Figure S10B). To determine the effect of exosomal miR‐27b‐3p on blood vessel permeability and tumour metastasis in vivo, we administered exosomes derived from EMT‐HCT116 cells and parental cells intratumorally into HCT116 xenografts. As shown in Figure 6F, more distant metastasis was detected in EMT‐HCT116‐exosomes treatment group, accompanied by increased miR‐27b‐3p expression in the tumours (Figure S10C). Collectively, these data indicate that CRC cells secrete exosomal miR‐27b‐3p to increase the in vivo vascular permeability and ultimately induce CTCs‐mediated metastasis.

### 2.7 Exosomal miR‐27b‐3p is associated with CRC progression and metastasis

**FIGURE 7:**
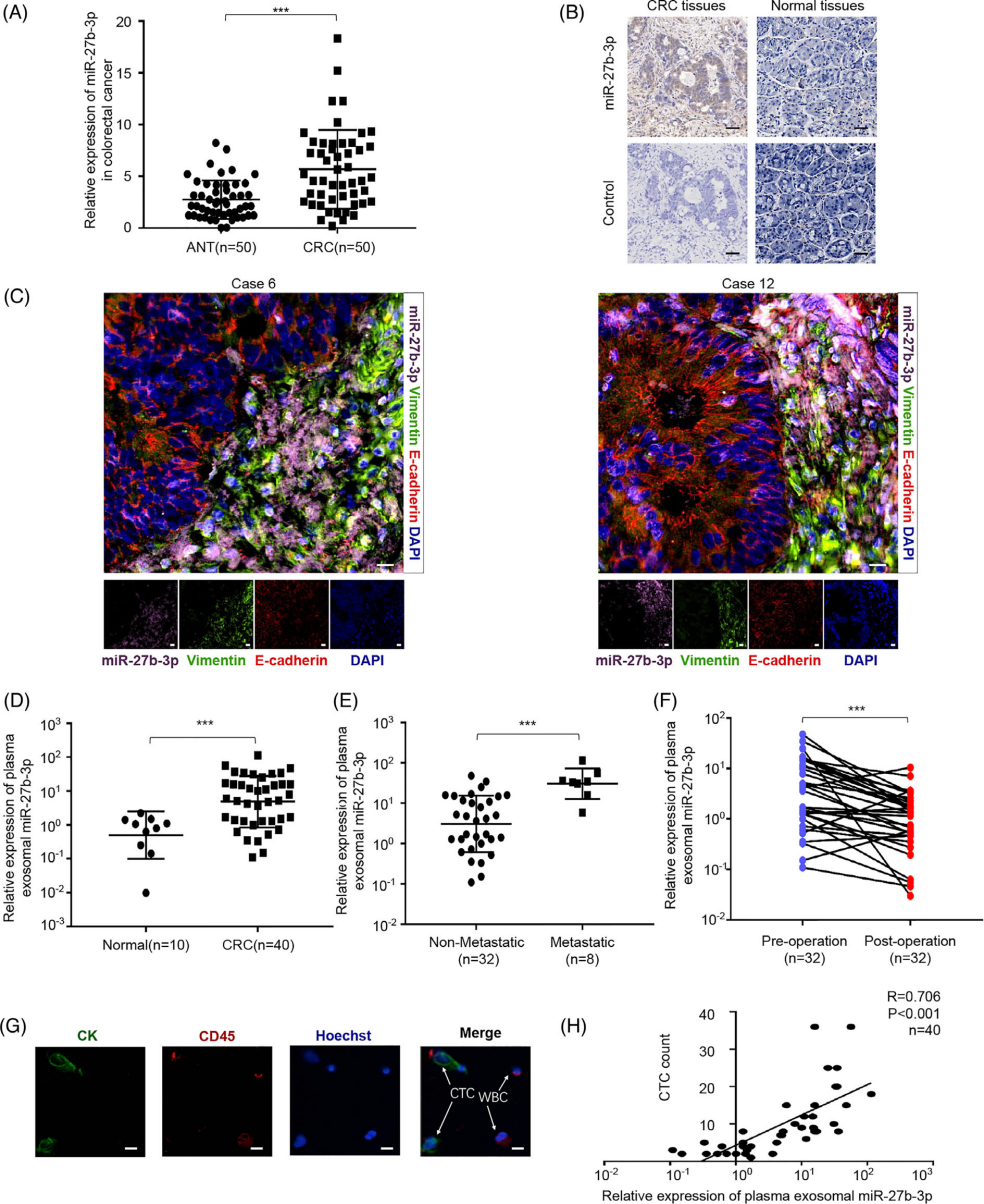
Plasma exosomal miR‐27b‐3p is associated with colorectal cancer (CRC) progression and metastasis. (A) The relative expression level of miR‐27b‐3p was measured with RT‐PCR in 50 pairs CRC samples. Error bars, SD. (B) Representative in situ hybridization (ISH) staining for miR‐27b‐3p expression in tissue microarrays. Scale bar, 40 μm. (C) Representative fluorescence in situ hybridization/ immunofluorescence (FISH/IF) staining for the distribution of miR‐27b‐3p, E‐cadherin, Vimentin in invasive front and normal colon tissue (*n* = 45). Scale bar, 20 μm. (D) The relative expression of exosomal miR‐27b‐3p in heathy donor (*n* = 10) and CRC patients (*n* = 40). (E) qRT‐PCR analysis of exosomal miR‐27b‐3p level in 40 cases of the CRC patients with or without metastasis (32 cases without metastasis, eight cases with metastasis). (F) qRT‐PCR analysis of exosomal miR‐27b‐3p level in matched plasma from CRC patients pre‐ and post‐operation (*n* = 32). (G) Representative images of circulating tumour cells (CTCs) from included patients. Scale bar, 20 μm. (H) Plasma exosomal miR‐27b‐3p is associated with CTCs count. Linear correlation of CTCs count with serum exosomal miR‐27b‐3p in CRC patients, respectively (*n* = 40). *p < .05, **p < .01, ***p < .001

**FIGURE 8:**
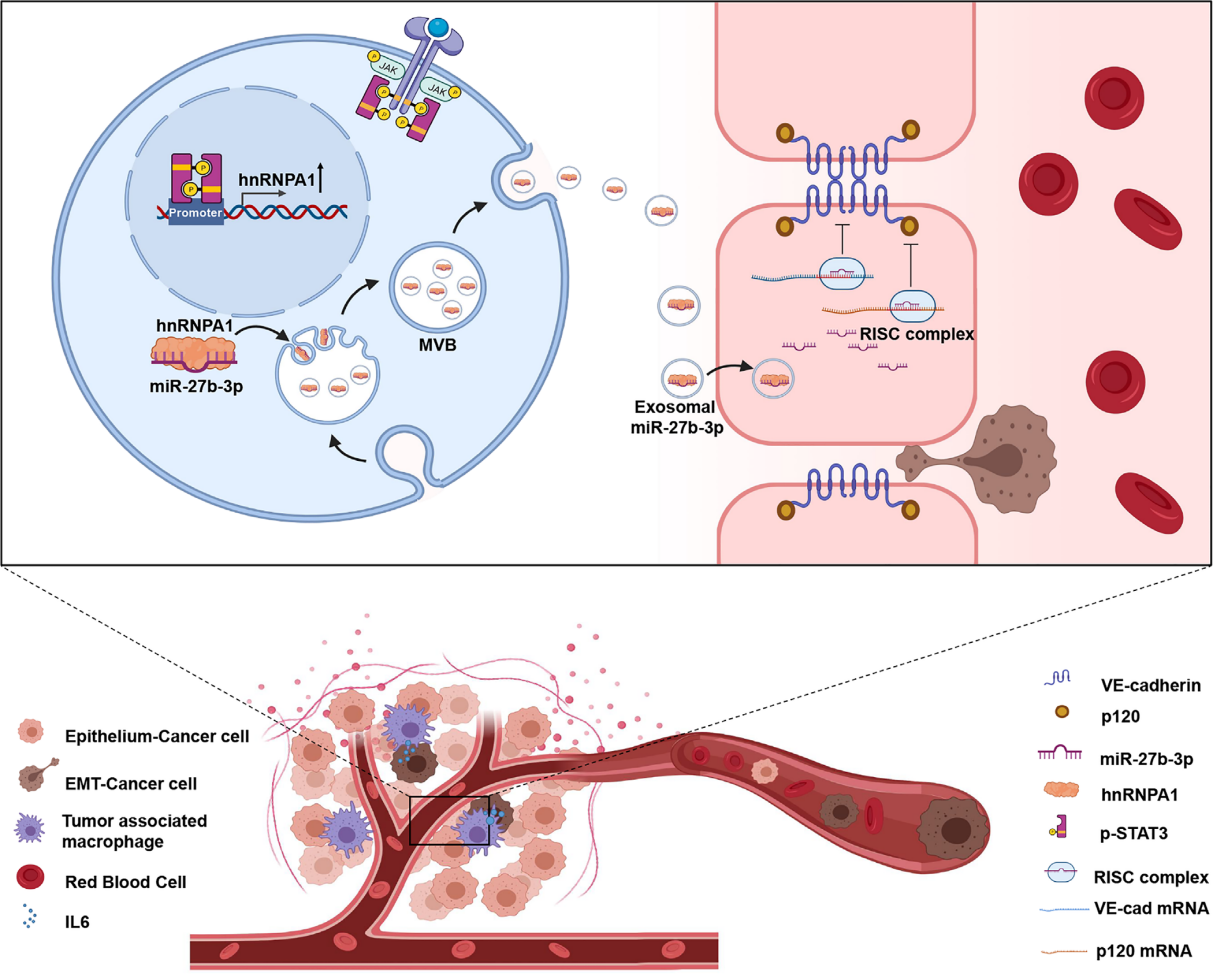
A model of exosomal miR‐27b‐3p functions in colorectal metastasis. Our study illustrated an intercellular communication between mesenchymal cancer cells and blood vessel endothelium. IL6, a pro‐inflammatory cytokine, binds to the IL6 receptor (IL6R) that sequentially phosphorylates STAT3 and promotes the EMT program. Otherwise, p‐STAT3 up‐regulated the expression of hnRNPA1, which packed miR‐27b‐3p into exosome and delivered into blood vessel endothelium cells. miR‐27b‐3p inhibits the level of adherent junction protein vascular endothelial cadherin (VE‐Cad) and p120 thus attenuates blood vessel barrier and facilitates circulating tumour cells (CTCs) generation and metastasis

To further investigate the oncogenic role of miR‐27b‐3p in CRC metastasis, we evaluated the level of miR‐27b‐3p in CRC tissues. A qRT‐PCR analysis of 50 pairs of CRC tissues (cohort 1) revealed a higher miR‐27b‐3p level (Figure 7A). Next, we conducted an extensive CRC tissue microarray consisting of 45 pairs of samples (cohort 2) along with an in situ hybridization (ISH) assay. The up‐regulation of miR‐27b‐3p was confirmed (Figure 7B, Figure S11A). Furthermore, we found that miR‐27b‐3p was mainly detected at the invasive front and closely correlated with EMT markers (Figure 7C, Figure S11B). We conducted qRT‐PCR to evaluate the level of exosomal miR‐27b‐3p, and after analyses, we found that exosomal miR‐27b‐3p level was closely correlated with miR‐27b‐3p level in the primary CRC tissues (Figure S11C). We next assessed the clinical significance of exosomal miR‐27b‐3p expression level in CRC patients. As compared with healthy donors, CRC patients had significantly higher exosomal miR‐27b‐3p levels (Figure 7D). Among the 40 CRC patients, metastatic patients had up‐regulated exosomal miR‐27b‐3p (Figure 7E). The plasma exosomal miR‐27b‐3p level decreased significantly after tumour resection, indicating that the tumour was the primary source of miR‐27b‐3p in plasma (Figure 7F). The Pearson's correlation analysis revealed a positive correlation between the plasma exosomal miR‐27b‐3p and the CTC count in CRC patients (Figure 7G,H). Further analysis indicated that the exosomal miR‐27b‐3p level in plasma was correlated with lymphovascular invasion (*p* < .01), deep tumour invasion (*p* < .05), lymph node metastasis (*p* < .05), advanced tumour stage (*p* < .05), and positive pre‐operative CTCs status (*p* < .01) in CRC patients (Table 1). In sum, these results suggested that miR‐27b‐3p is aberrantly expressed in both CRC tumour tissues and plasma exosomes, and elevated exosomal miR‐27b‐3p in plasma is correlated with tumour invasion and metastasis. These findings indicate that the exosomal miR‐27b‐3p may become a promising biomarker for CRC metastasis (Figure 8).

**TABLE 1:** **Relationship between serum exosomal miR‐27b level and clinicopathological features in** colorectal cancer (CRC) **patients**

	Number of patients		miR‐27b‐3p expression			
Parameters	Number	%	<median	≥median	χ^2^ value	*p* value
Gender						
Male	19	47.5	7	12	2.506	.1134
Female	21	52.5	13	8		
Age, years						
<60	14	35	8	6	.440	.5073
≥60	26	65	12	14		
Tumour site						
Colon	24	60	11	13	.417	.5186
Rectal	16	40	9	7		
Tumour size, cm						
<5	22	55	10	12	.404	.5250
≥5	18	45	10	8		
Tumour grade						
Moderate/well	18	45	11	7	1.616	.2036
Poor	22	55	9	13		
Lymphovascular invasion						
Absence	19	47.5	15	4	12.130	<.001^***^
Presence	21	52.5	5	16		
Perineural invasion						
Absence	16	40	7	9	.417	.5186
Presence	24	60	13	11		
Tumour invasion						
T1‐2	18	45	13	5	6.465	.0110*
T3‐4	22	55	7	15		
Lymph node metastasis						
N0‐1	20	50	14	6	6.400	.0114*
N2‐3	20	50	6	14		
TNM stage^a^						
I/II	19	47.5	14	5	8.120	.0044**
III	21	52.5	6	15		
CEA, ng/ml						
<5	22	55	12	10	.404	.5250
≥5	18	45	8	10		
Preoperative CTCs status						
Negative	17	42.5	13	4	8.286	.0040**
Positive	23	57.5	7	16		

Abbreviations: CEA, carcinoembryonic antigen; CTCs, circulating tumour cell; TNM, tumour‐node‐metastasis.

* p < .05.

** p < .01.

^a^ The 8th edition of the American Joint Committee on Cancer (AJCC) Cancer Staging Manual.

## 3 DISCUSSION

The blood vascular integrity is a barrier against the generation and movement of CTCs. In this study, we demonstrated that miR‐27b‐3p is markedly up‐regulated in exosomes derived from EMT CRC cells, which can be further delivered into blood vessel endothelial cells. Exosomal miR‐27b‐3p attenuated VE‐Cad and p120, disrupting vascular permeability and facilitating CRC cell intravasation, CTC generation and ultimately metastasis. Furthermore, we found that exosomal miR‐27b‐3p level in plasma was associated with CTC count. Overall, our work demonstrated the function and clinical significance of secreted miR‐27b‐3p in CRC metastasis.

The interaction between cancer cells and TME plays significant role in metastasis, enhancing the intravasation of cancer cells by inducing EMT and promoting permeability of blood vessels.^34^ IL6 is one of the most abundant inflammatory mediators in TME and is reported to promote EMT and subsequent tumour progression and metastasis.^35^, ^36^ Herein, we revealed that inflammatory cytokine IL6‐triggered EMT significantly increases tumour blood vessel permeability by specifically increasing the packaging of miR‐27b‐3p in cancer‐derived exosomes. Several studies reported that highly invasive cancer cells remodelled TME vascular permeability and enhanced distant metastasis. Zhou et al. reported that metastatic breast cancer cells secrete miR‐105 through exosomes to facilitate metastasis by increasing blood vessel permeability.^20^ Haidari et al. suggested that invasive cancer cells enhance vascular permeability by inducing phosphorylation of VE‐Cad.^37^ Our findings are consistent with those of previous studies. To the best of our knowledge, the present study is the first study reporting how cancer cells alter vascular permeability of TME after undergoing EMT. We previously reported that TAM‐derived pro‐inflammatory cytokines induce EMT and CTCs‐mediated metastasis in invasive front.^12^, ^13^ Taken together, we discuss how the EMT process of tumour cells is activated in TME and the mechanism by which EMT tumour cells alter the TME blood vessel permeability in the TME. These interactions collaboratively promote CTCs‐induced metastasis.

Exosomes are an important mediator of intercellular communication,^38^ and miRNA can be transferred through exosomes between tumour cells and blood vessel cells. As per studies, exosome‐mediated miR‐105, miR‐25‐3p and miR‐103 promote tumour progression and metastasis by targeting tight junction proteins and altering blood vessel permeability.^19^, ^20^, ^39^ The reported functions of miR‐27b‐3p in cancer progression include promoting proliferation and invasion.^40^, ^41^, ^42^ We found that CRC‐derived exosomal miR‐27b‐3p facilitates metastasis by enhancing TME vascular permeability. These results suggested that in CRC, miR‐27b‐3p is a multi‐functional oncogene. Noticeably, in CRC, serum miR‐27b‐3p elevates in CRC patients and decreases after surgical removal of tumours; furthermore, miR‐27b‐3p level in plasma associated with shorter PFS in metastatic CRC patients.^43^, ^44^ These studies reveal a correlation between secreted miR‐27b‐3p and the CRC patients prognosis. However, the exact role that exosomal miR‐27b‐3p plays in CRC progression and metastasis has remained unclear. And our findings suggested that exosomal miR‐27b‐3p is up‐regulated after EMT and increases vascular permeability by targeting VE‐Cad and p120. VE‐cad is essential to the organization of adherent junction since it maintains the junction's integrity and inhibits unrestrained vascular growth.

Meanwhile, p120 is linked with the cytoplasmic domain of VE‐Cad and thus facilitates the stabilization of VE‐Cad.^17^ miR‐27b‐3p inhibited tumour vasculogenic mimicry inside the ovarian cancer cells by targeting VE‐Cad.^45^ Integrating exosome miRNA microarray analysis, miRNA target prediction in silico and functional analysis, we revealed that exosomal miR‐27b‐3p in HUVECs down‐regulates VE‐Cad and p120, and subsequently promotes vascular permeability.

Given the specific and sophisticated mechanisms involved in exosomal content packaging, understanding the underlying mechanism of up‐regulation of exosomal miR‐27b in EMT CRC cells is essential. hnRNPA1 is involved in the transmission of the miRNA into exosomes.^24^, ^32^, ^46^This study revealed that hnRNPA1 binds with miR‐27b‐3p and mediates its transmission into exosomes, and silenced hnRNPA1 resulted in a lower miR‐27b‐3p level in mesenchymal CRC exosomes rather than altering the endogenous miR‐27b‐3p level. These results demonstrate that RNA in exosomes is not a simple reflection of the RNA composition of producer cells but rather involves a highly selective process.^25^, ^26^ However, hnRNPA1 may also contribute to other factors into exosome in EMT CRC cells which can regulate tumour blood vessel permeability, and how hnRNPA1 mediated the package into EMT‐CRC cells‐derived exosomes is still need fully elucidated. Interestingly, Yao et al. reported that STAT3 could transcriptionally increase the expression level of hnRNPA1.^33^ STAT3, widely and aberrantly activated in many tumours, including CRC, is involved in cell growth, EMT and angiogenesis.^47^, ^48^, ^49^ We also found that STAT3 transcriptionally up‐regulates the expression of hnRNPA1. STAT3 directly binds on two sites in the hnRNPA1 promoter region. Furthermore, inhibiting STAT3 decreased miR‐27b‐3p levels in mesenchymal tumour cell‐derived exosomes. The STAT3/hnRNPA1 pathway can partially explain the up‐regulation of miR‐27b‐3p in EMT cancer cell‐derived exosomes. Again, we highlighted the pivotal role of STAT3 in cancer progression and metastasis. We propose that STAT3/hnRNPA1‐mediated miR‐27b‐3p exosome packaging may be a therapeutic target for eliminating the circulating miR‐27b‐3p in CRC.

## 4 CONCLUSION

In conclusion, our findings revealed that EMT CRC cell‐derived exosomal miR‐27b‐3p that is transferred to endothelial cells enhances blood vessel permeability and induces CTCs generation by targeting VE‐Cad and p120. Upstream STAT3 up‐regulated hnRNPA1 by binding to its promoter, and hnRNPA1 mediated the packaging of miR‐27b‐3p. Our study highlighted that STAT3/hnRNPA1/miR‐27b‐3p signal cascade and exosomal miR‐27b‐3p might be promising diagnostic biomarkers for CRC metastasis.

## 5 MATERIALS AND METHODS

### 5.1 Patients and clinical specimens

CRC specimens were obtained from patients after pathological diagnosis at Zhongnan Hospital of Wuhan University (Wuhan, China). Five‐millilitre peripheral blood samples were amassed during hospitalization to detect CTCs. Moreover, patients, who underwent neoadjuvant chemotherapy/radiotherapy, were excluded. The entire samples were collected from patients with consent. The Research Ethics Committee of Wuhan University (Wuhan, Hubei, China) approved this study.

### 5.2 Cell culture and reagents

Normal intestinal epithelium cell line NCM460 and five CRC cell lines (LOVO, HCT‐116, DLD‐1, SW620 and SW480) were bought from the Chinese Academy of Sciences. NCM460 and CRC cell lines were cultured in 37°C incubator with 5% CO_2_ using Roswell Park Memorial Institute (RPMI) 1640 medium (Gibco, USA) containing 10% fetal bovine serum (FBS). Human umbilical vein endothelial cells were purchased from the American Type Culture Collection. Cells were cultured in M199 medium (Invitrogen, Shanghai, China) containing 10% FBS (Gibco, USA). PBS containing .1% BSA was used to dissolve recombinant human IL6 (R&D Systems) to a final concentration of 50 ng/ml. Exosomes were pre‐incubated with 2 μg/ml annexin V (Biosolar, China) for 2 h before culture with HUVECs.

### 5.3 Exosomes isolation, characterization and treatment

CRC cells were cultured in RPMI‐1640 medium containing with 10% exosome‐free FBS. FBS was depleted of exosomes by ultracentrifugation using Beckman Optima L‐100XP (Beckman Coulter, USA) at 110 000 × g at 4°C overnight. Cell culture medium was collected after 72 h and centrifuged at 2000 g for 20 min and 2 5000 g for 40 min at 4°C. The supernatant was filtered with a .22 μm filter and centrifuged at 110 000 × g for 90 min at 4°C. Harvested exosomes were quantified by the BCA Protein Assay kit (Millipore, Billerica, MA, USA). Exosomes were observed and identified on transmission electron microscopy HT7700 (HITACHI, Japan). Exosomes were labelled with PKH67 (Sigma, St. Louis, MO, USA) as previously described.^50^ The exosomes were added with 10 ml PBS, collected by ultracentrifugation at 110 000 × g for 90 min, then resuspended in PBS and then incubated with HUVEC for 240 min and photographed by a fluorescence microscope. For cell processing, the number of recipient cells was controlled to 2 × 10^5^ and exosomes and recipient cells were incubated for 72 h. CM of EMT‐HCT116/SW620 cells treated with RNase (2 mg/ml) alone or with Triton X‐100 (.1%)

### 5.4 Animal experiment

All procedures of animal experiments were executed according to guidelines of Hubei Provincial Key Laboratory of Tumor Biological Behavior. The Animal Health and Ethics Committee of Zhongnan Hospital of Wuhan University also approved all operations of animal experiments. In order to perform the orthogonal heterosexual transfer assay, 5 × 10^6^ CRC cells were injected subcutaneously on the right ventral area of female BALB/c nude mice (4‐6 weeks). Once the xenografts were established (within 14 days), the xenograft tumours were dissected under asepsis and the fibrotic tissue was separated from tumour, excised and minced tumour tissue into 1 mm^3^ pieces. Under anaesthetization, both the caecum and ascending colon of the nude mice were exteriorized, and the 1 mm^3^ pieces of xenograft were implanted sub‐serosally. After suture, the bowel was returned into abdomen. For permeability assay, rhodamine–dextran (100 mg/kg) was injected into the tail vein of mice. To detect the CTCs and exosomal miR‐27b‐3p in the blood of nude mice, the blood after cardiac puncture and centrifugation was collected with ethylene diamine tetraacetic acid, and the blood volume was 1 ml. Finally, the cells and plasma are separated. The cells were resuspended by PBS for further CTCs detection. Exosomes from plasma were further isolated with exoRNeasy Serum/Plasma MaxiKits (QIAGEN, Germany). The RNA was separated from exosome by using Trizol reagent, and qRT‐PCR was applied to quantify the miR‐27b‐3p level in exosome. The paraffin‐embedded lungs and the livers were serial sectioned and after hematoxylin‐eosin (HE) staining, they were observed through a microscope.

### 5.5 Tube formation assay, angiogenesis assay, endothelial permeability assay

For tube formation analysis, Matrigel was placed in a German ibidi plate (approximately 10 microliters per well) and incubated in incubator to polymerize. Inoculate the treated HUVEC in the Matrigel‐coated wells. Tube formation was observed with a microscope at 12 h. The angiogenesis capacity is determined by calculating the number of measuring tubes. The experiment was repeated independently three times. Matrigel was placed in a German ibidi plate (approximately 10 microliters per well) and incubated for 30 min to polymerize Matrigel. For Permeability assay, a single layer of treated HUVEC was grown on a .3 cm^2^ polyethylene terephthalate ultrafilter (pore size .4 μm; BD Biosciences; Franklin Lakes, NJ). EVOM2 voltmeter 17 (World Precision Instruments; Sarasota, FL) was used to calculate the unit area resistance. The permeability of the treated HUVEC monolayer membrane (average pore size .4 μm; BD Biosciences) was evaluated by the transfer of rhodamine–dextran (average MW ∼ 70,000; Sigma). Rhodamine–dextran was added to the top well at a concentration of 20 mg/ml, excited at 544 nm and emitted at 590 nm using a SpectraMax microplate reader, measuring 40 μl medium aliquots in a time course. GFP‐labelled HCT116 in the lower chamber was calculated after 24 h.

### 5.6 RNA isolation and quantitative real‐time PCR

Following the protocol, RNA was extracted with Trizol reagent (Vazyme, China). The reverse transcription of RNA into cDNA uses RT Master kit (Vazyme, China). The obtained cDNA was used for quantitative real‐time PCR reaction with SYBR‐Green PCR Master Mix (Vazyme, China).

### 5.7 Western blot

Proteins were seperated by SDS‐PAGE gel and transfered to the polyvinylidene fluoride (PVDF) membrane (Millibo, USA). After blocking with 5% skim milk, incubate at 4°C with primary antibody overnight. Place the washed membrane in the secondary antibody, and incubate at room temperature for 60 min. Bio‐Rad ChemiDoc XRS was used to detect protein + system. Density analysis using Bio‐Rad Image Lab software.

### 5.8 Transendothelial invasion assay

Cell invasion assay using 24 wells Transwell (pore size 8 μm; Corning) was pre‐coated Matrigel (Falcon 354480; Corning). Matrigel (5 mg/ml) was purchased from Corning and stored in ‐20℃, and before transwell assay, the Matrigel was placed in 4℃ overnight and diluted in RPMI1640 with ratio 1:10; the upper compartment was precoated with 50‐μl diluted Matrigel and incubated at 37℃ for 2 h to allow the Matrigel solidification. A total of 1 × 10^5^ cells were suspended in 500 μl RPMI 1640 with 1% FBS and added to the upper chamber, while 750 μl RPMI 1640 contains 10% Place FBS on the bottom for 48 h. Count and take pictures under five optical microscope fields (×100 magnification). Every experiment is repeated three times. Transendothelial invasion test was functioned to discover GFP‐expressing HCT‐116 cells that were invaded by HUVEC monolayer with or without exosome treatment.

### 5.9 Wound healing assay

For wound‐healing assays, cells grow to 90%–100% confluence six‐well plate; the wound is scratched by plastic tips. In order to remove cell debris, the main cells are washed three times in PBS. The time to take photos of the migrating cells in the front of the wound was selected 24 h later. All experiments were repeated three times.

### 5.10 CTCs isolation and identification

CTCs were enrichment and identification as previously described.^12^

### 5.11 Transfection of miR‐27b‐3p mimic, inhibitor and siRNA of the target genes

STAT3/hnRNP A1 siRNA was obtained from GenePharma (Shanghai, China). Purchase hsa‐miR‐27b‐3p mimic, inhibitor (namely anti‐miR‐27b‐3p) and corresponding NC through RiboBio(Guangzhou, China). Wash cells (PBS, pH 7.4) during preparation for transfection. The Lipofectamine 2000 was applied to facilitate transfection (Invitrogen, USA); the final concentration of transfection is 50 nM.

### 5.12 RIP assay and ChIP assay

RIP assays were performed using a RIP kit (Millibo, Massachusetts, USA). Briefly, the collected cells (the number of cells in each group is about 5 × 10^7^) was lysed in lysis buffer and the supernatant was co‐incubation with specific antibody, after incubation, the antibody was digested and the RNA was extracted with Trizol. Isolated miRNA is reverse transcribed and then detected by real‐time PCR. The kit used for ChIP analysis is Cell Signaling SimpleChIP Enzyme Chromatin IP (#9003, USA). After elution and reversal of cross‐links, qRT‐PCR was applied to quantify the precipitated DNA; primers for CHIP1, CHIP2 and NC were listed in Supporting Table.

### 5.13 Fluorescence in situ hybridization and in situ hybridization

FISH was performed in tissue sections by using FISH kit (Bosterbio, USA) separately according to the manufacturer's protocol. The miR‐27b‐3p detection probe was synthesized by Bosterbio (Bosterbio, USA).

### 5.14 Immunofluorescence and immunohistochemistry staining

Tissue sections (4 μm thick) or cells grown on cover slips were fixed with formalin (containing .1% diethylpyrocarbonate) for 20 min. Cells were washed with PBS. After permeabilization with .4% pepsin for 5–120 s, blocking buffer (3% BSA) was added. Samples were then incubated with primary antibodies, followed stain with secondary antibody. Nuclei were stained with 4',6‐diamidino‐2‐phenylindole. Section and cell were observed, and images were attained using an Olympus BX53 fluorescence microscope. VE‐Cad, p120, E‐Cad and Vimentin expression were quantified as previously described.^51^

### 5.15 Plasmid constructs

The genomic sequences of human VE‐Cad and p120‐3′‐UTR were amplified by PCR and cloned into the XhoI/NotI site of pmiR‐RB‐Report luciferase reporter vector (RiboBio, Guangzhou, China); their corresponding mutant (Mut) 3′‐UTR sequences were generated by site‐directed PCR‐mutagenesis and inserted into the same sites of pmiR‐RB‐report luciferase reporter vector. All genomic products were confirmed by sequencing. Primers for this experiment are listed in Supporting Table.

### 5.16 Luciferase assay

In briefly, HEK‐293T was co‐transfected with wild or mutant luciferase reporter plasmid and mimics of miR‐27b‐3p (miR‐RiboTM NC). Forty‐eight hours after co‐transfection, the luciferase activity was determined with the dual‐luciferase reporter assay system (Promega, USA). For the miRNA promoter assay, a luciferase reporter plasmid containing the full‐length miR‐27b‐3p promoter was constructed and inserted into tumour cells with or without the co‐transfection of VE‐Cad/p120. The luciferase activity was determined after 48 h. Every reaction was conducted in triplicate.

### 5.17 Statistical analysis

Three independent biological replicates were carried out for cell culture experiments. Unless otherwise specified, all experimental data provided were expressed by means. All statistical analyses used GraphPad Prism software (version 6.0, GraphPad software, United States) and SPSS statistical software (version 22.0, IBM SPSS, United States). Two‐tailed Student's tests were used to compare the means quantitative data between groups, or when more than two groups were compared by one‐way analysis of variance. Values of *p* < .05 are determined statistically significant.

## CONFLICT OF INTEREST

The authors declare that they have no conflict of interest.

## Supporting information

SUPPORTING INFORMATION

## Data Availability

The data of RNA sequencing generated in this study are available in the SRA database: SRP227899. Other data and material are available at the Clinical and Translational Medicin's website.
