# Supplementary material for: EMT‐cancer cells‐derived exosomal miR‐27b‐3p promotes circulating tumour cells‐mediated metastasis by modulating vascular permeability in colorectal cancer
Source: Clin Transl Med. 2021 Dec 22;11(12):e595. doi: 10.1002/ctm2.595 (PMC8694332; doi:10.1002/ctm2.595)
Supplement: Supplementary file 1 — SUPPORTING INFORMATION [file CTM2-11-e595-s001.pdf]

Supplementary Materials for

Cancer cells derived exosomal mir-27b-3p promotes circulating tumor cell mediated metastasis by modulating vascular permeability in colorectal cancer

Rongzhang Dou[1,2,3,4*] , Keshu Liu[1,2,3,4*] , Chaogang Yang[1,2,3,4*] , Jinsen Zheng[1,2,3,4*] , Dongdong Shi[1,2,3,4] , Xiaobin Lin[5] , Chen Wei[6] , Chunxiao Zhang[1,2,3,4] , Yan Fang[1,2,3,4] , Sihao Huang[1,2,3,4] , Jialin Song[1,2,3,4] , Shuyi Wang[1,2,3,4] , and Bin Xiong[1,2,3,4]

[*]These authors contributed equally to this work.

Correspondence: Dr. Bin Xiong, Department of Gastrointestinal Surgery, Zhongnan Hospital of Wuhan University, Hubei Key Laboratory of Tumor Biological Behaviors, Hubei Cancer Clinical Study Center, Donghu Road 169, Wuhan 430071, Hubei, China.

E-mail: binxiong1961@whu.edu.cn

Correspondence: Dr. Shuyi Wang, Department of Gastrointestinal Surgery, Zhongnan Hospital of Wuhan University, Hubei Key Laboratory of Tumor Biological Behaviors, Hubei Cancer Clinical Study Center, Donghu Road 169, Wuhan 430071, Hubei, China.

E-mail: shuyiwang@whu.edu.cn

Financial support: This work was supported by grants from the National Natural Science Foundation of China (81872376), National Natural Science Fund Youth Fund of China (81702411), Zhong nan Hospital of Wuhan University, Technology and Innovation Seed Found (znpy2016058).

Conflict of interest disclosure statement: The authors declare no potential conflicts of interest.

S1A

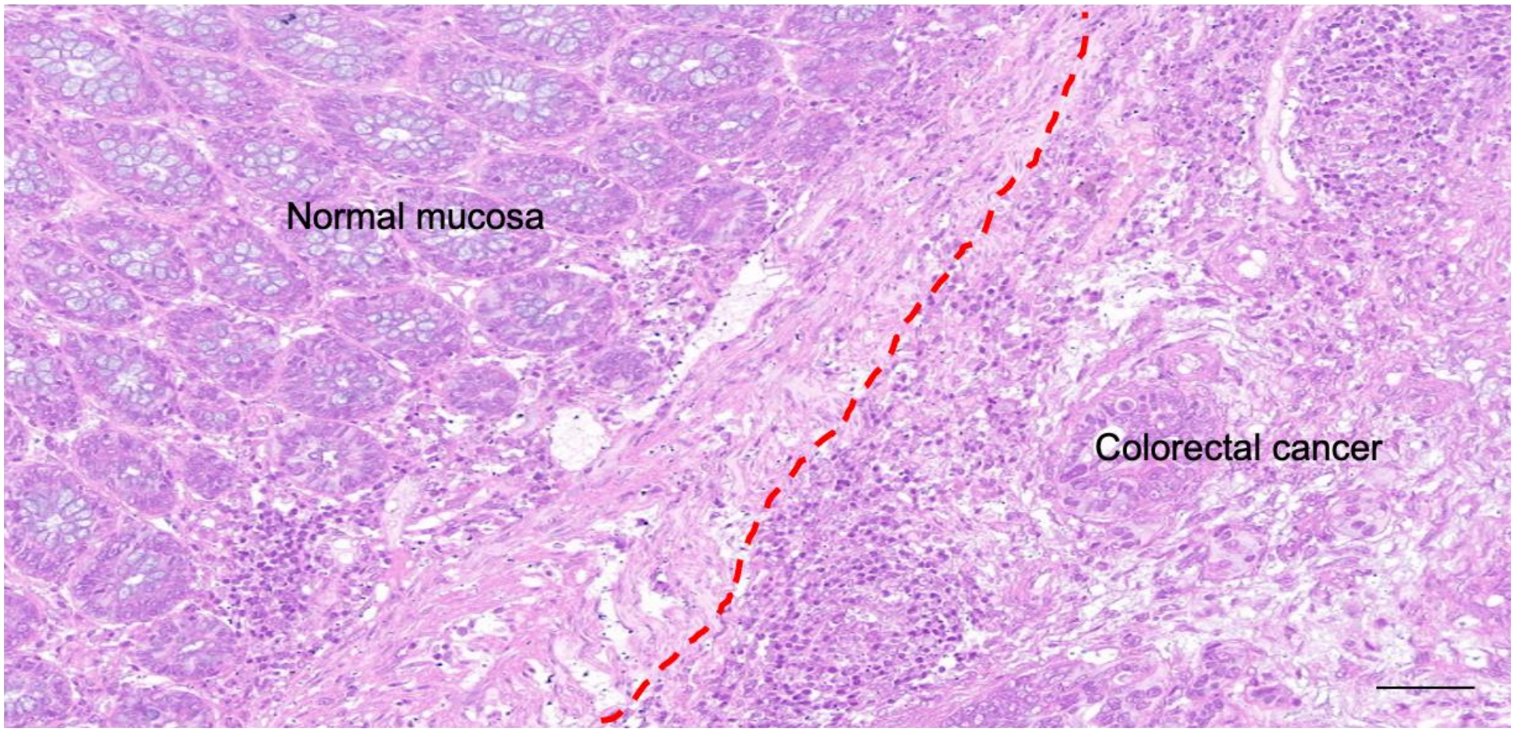

Normal mucosa
Colorectal cancer

S1B

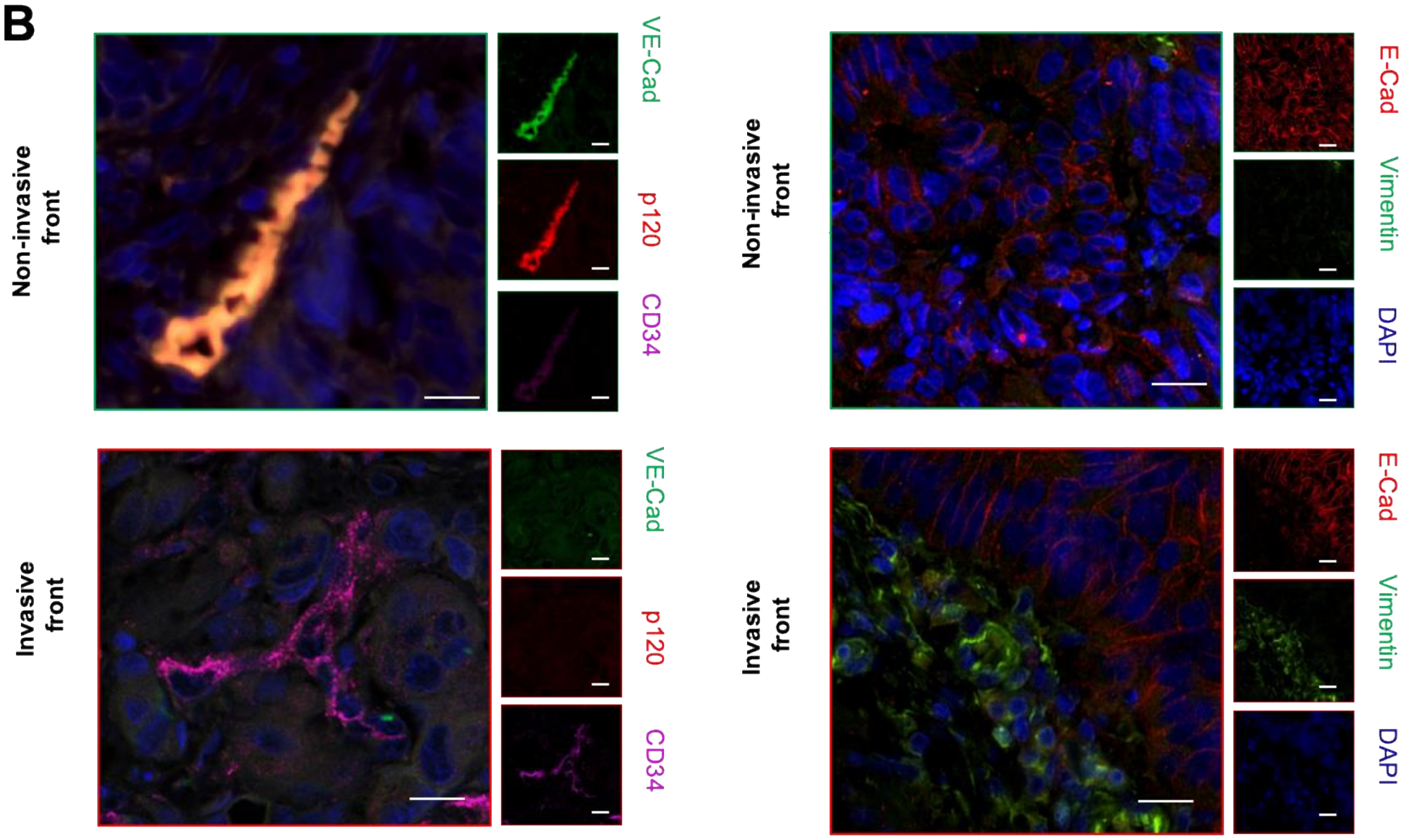

Non-invasive front
VE-Cad
p120
CD34
Invasive front
VE-Cad
p120
CD34
Non-invasive front
E-Cad
Vimentin
DAPI
Invasive front
E-Cad
Vimentin
DAPI

Supporting Fig.1

(A). Representative image of invasive front determined by HE staining from CRC specimens. Scale bar represents 50 μm.

(B). Representative immunofluorescence staining for CD34, VE-cadherin, p120 and EMT markers in the invasive front and non-invasive front of serial sections from included patient 3 and 15, respectively. Scale bar represents 20 μm.

S2A

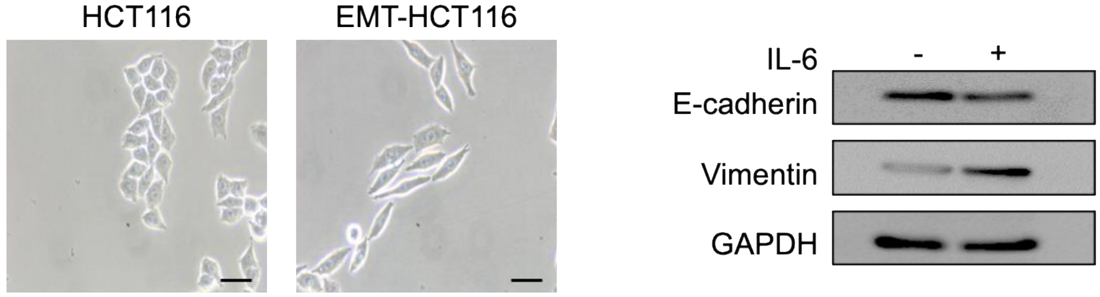

S2B

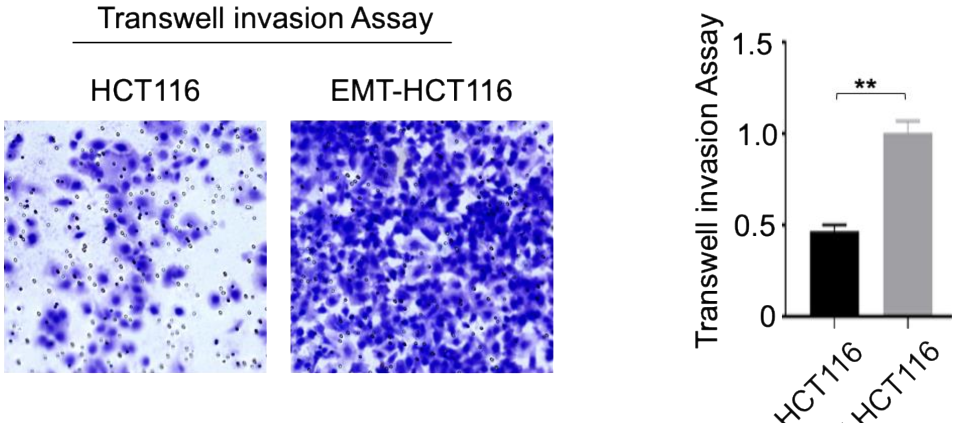

S2C

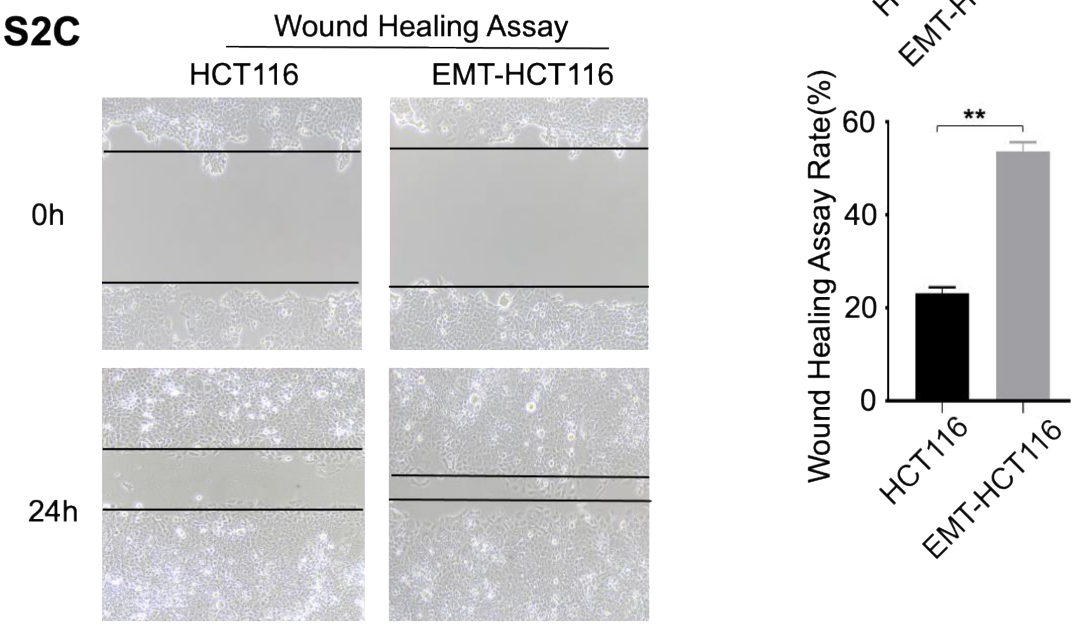

S2D

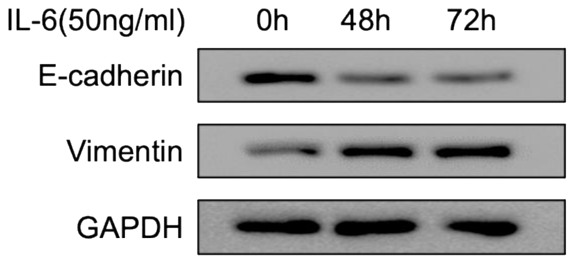

Supporting Fig.2 Transwell invasion assays and wound healing assay of HCT116 and EMT-HCT116 cells .

(A). Left panel: IL6 treatment led to a spindle-shaped morphology, loss of cell-to-cell contact in HCT116 cells. Scale bar represents 20 μm. Right panel: Expression of the E-cadherin and Vimentin proteins in colorectal cancer cell lines exhibiting epithelial（E）or mesenchymal（M）phenotypes was determined by Western blot .

(B). Transwell invasion assays of HCT116 and HCT116M cells . Total number of cells in five fields was counted manually Mean ± SD are shown. Statistical analysis was conducted using one-way ANOVA. (magnification, × 100) . Error bars, SD, ** $P < 0.01$.

(C). Photomicrographs and quantifications of wound healing assay.(magnification, × 40) . Error bars, SD, ** $P < 0.01$.

(D). Western blot was conducted to detect the expression of the E-cadherin and Vimentin proteins in HCT116 cell line in time points as indicated at the top.

S3A

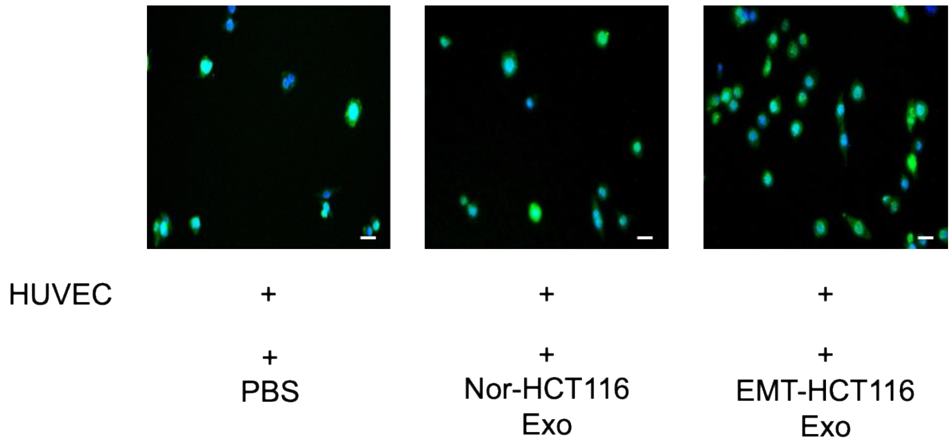

S3B

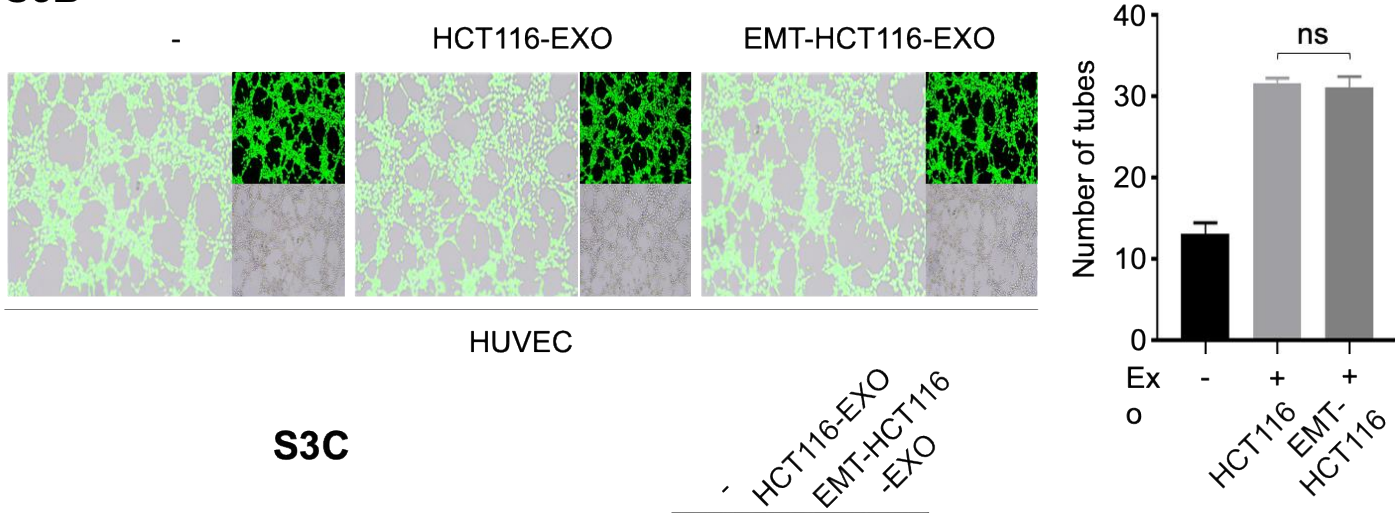

S3C

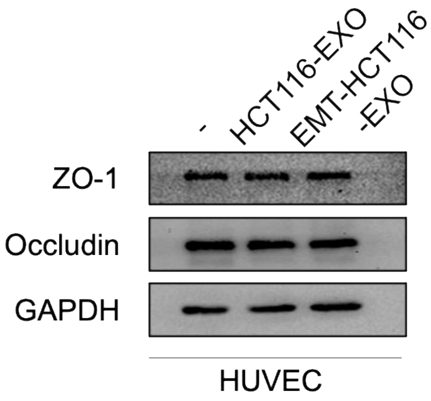

S3D

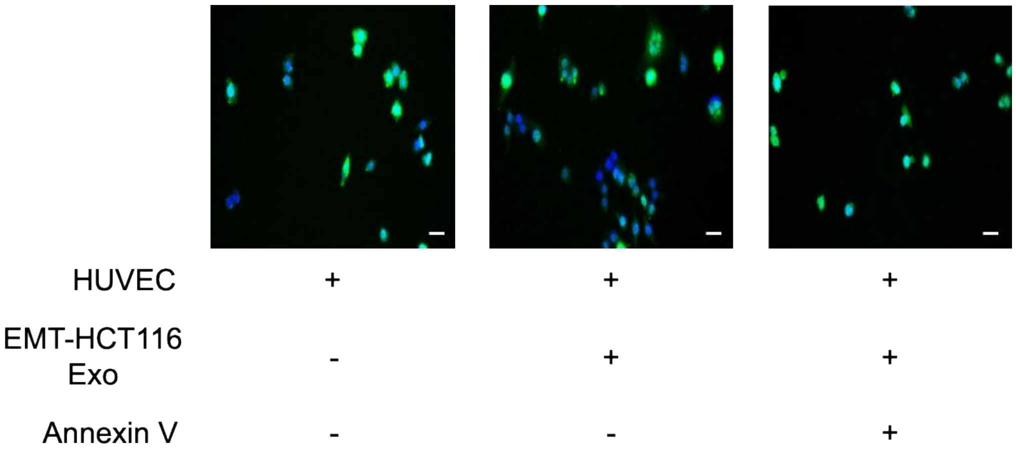

Supporting Fig.3

(A). Representative images for tumor cell invasion through the HUVEC monolayer. Scale bar represents 50 µm.

(B). 2-day tube formation ability was established, HUVECs were cultured with (+) or without (-) the CM derived from HCT116 or EMT-HCT116 cells for 48 hours. Scale bar represents 100 µm. Mean SEM are provided (n = 3). EMT-HCT116 cells has no obvious effect to enhance tube formation compared with HCT116-derived exosomes. Error bars, SD, ns=non-significan, magnification, × 100

(C). HUVECs were cultured without or with the exosomes derived from HCT116 or EMT-HCT116 cells. Expression of the ZO-1, Occludin proteins was determined by Western blot

(D). Representative images for tumor cell invasion through the HUVEC monolayer. Scale bar represents 50 µm.

S4A

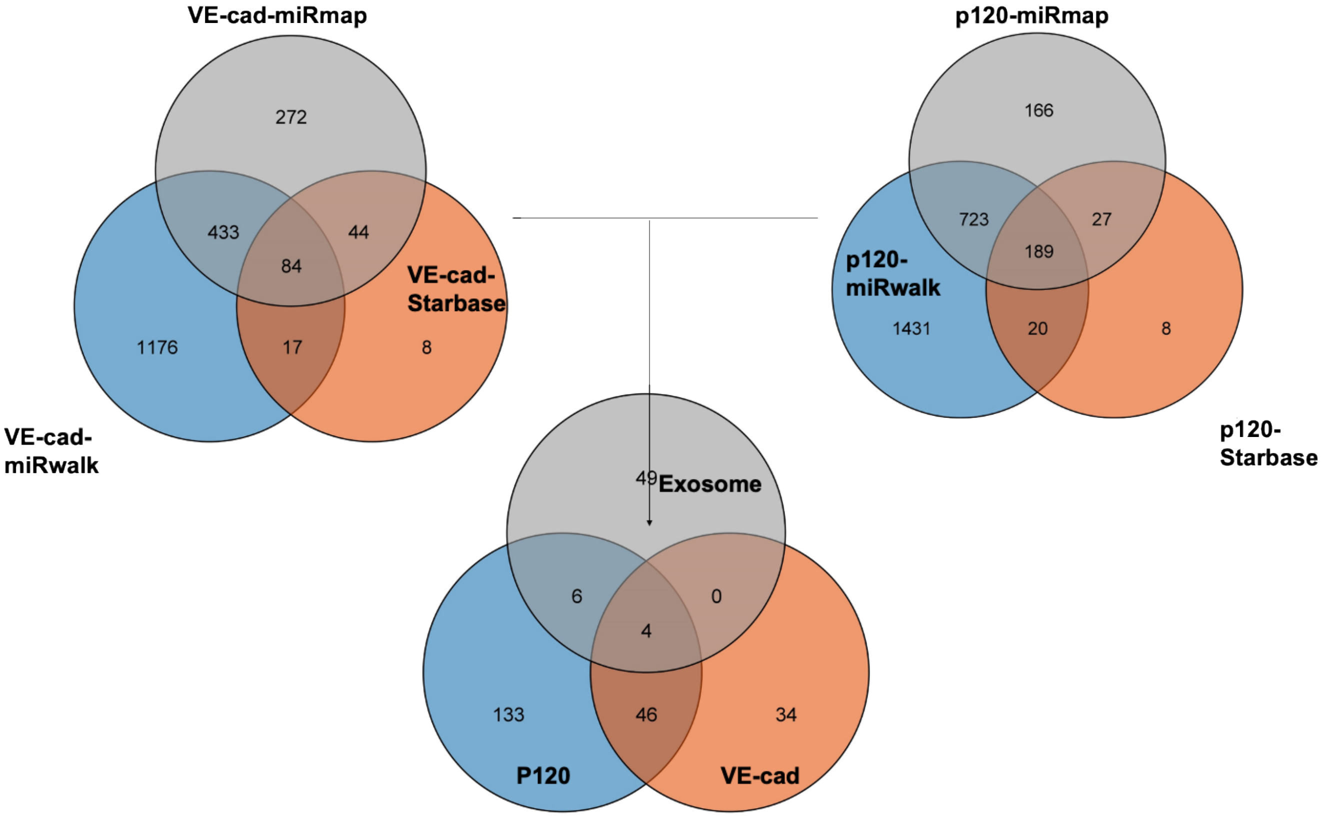

Poteneial miRNAs targeting VE-cad/P120(Predicted by 3 miRNA targeting databases)		
hsa-miR-140-5p	hsa-miR-27a-3p	hsa-miR-423-5p
hsa-miR-27b-3p		

S4B

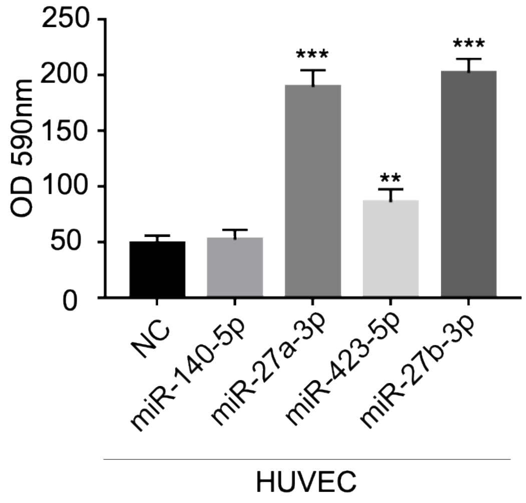

S4C

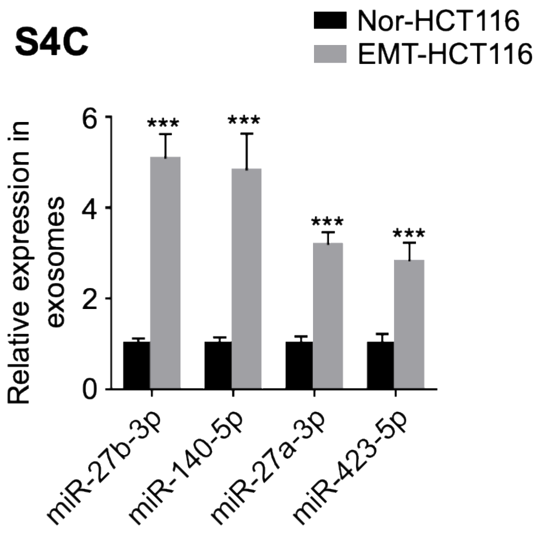

Supporting Fig.4

(A). Target miRNA prediction of VE-cadherin and p120 with TargetScan and miRWalk

(B). Transfect HUVEC with five candidate miR-mimics or negative control. Then add rhodamine-dextran to the upper chamber, and after incubating for 60 minutes, detect the content of dextran(OD 590 nm) in the lower chamber.

(C). RT-PCR analysis of four candidate miRNAs exosomal expression in Nor-HCT116 and EMT-HCT116.

S5A

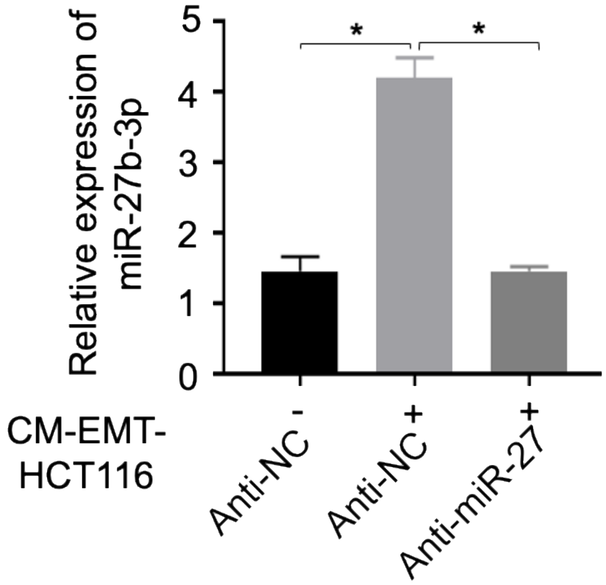

Relative expression of miR-27b-3p
5
4
3
2
1
0
*
*
CM-EMT-HCT116
-
+
+
Anti-NC
Anti-NC
Anti-miR-27

S5B

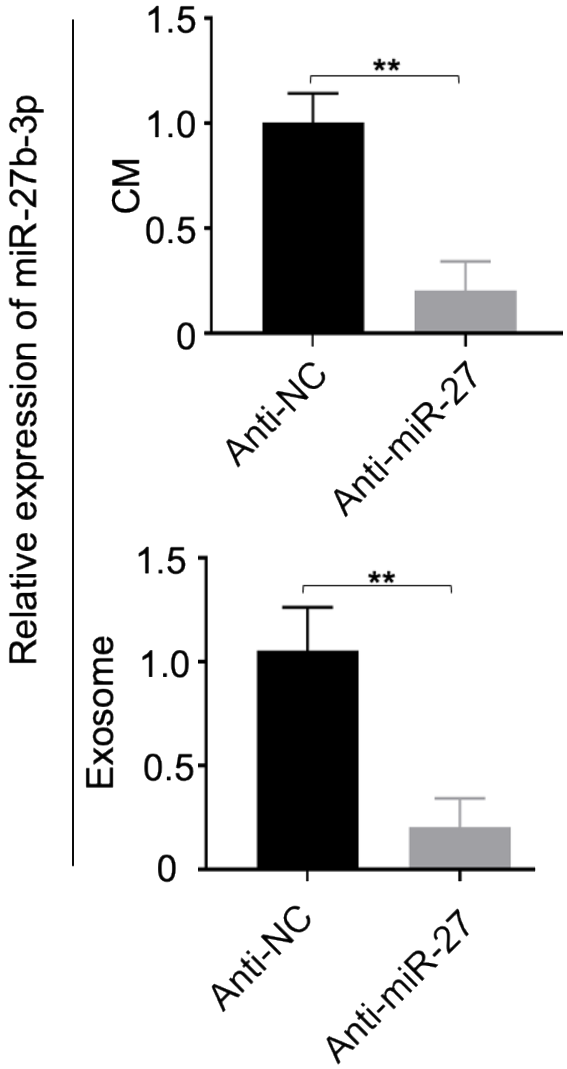

Relative expression of miR-27b-3p
CM
1.5
1.0
0.5
0
**
Anti-NC
Anti-miR-27
Exosome
1.5
1.0
0.5
0
**
Anti-NC
Anti-miR-27

Supporting Fig.5 The exosomes miR-27b-3p secreted by colorectal cancer cells are delivered into endothelial cell

(A). Anti-endogenous miR-27 in EMT-HCT116 cells attenuates the ability of EMT-HCT116 CM to increase miR-27 levels in the receptor HUVEC. HUVEC was cultured with or without exosomes derived from EMT-HCT116 cells transfected with anti-NC or anti-miR-27. Error bars, SD, * $P < 0.05$, ** $P < 0.01$, *** $P < 0.001$

(B). RT-PCR detection of anti-miR-27 transfected SW620 cells showed reduced miR-27 levels in cells, CM and exosomes. Error bars, SD, ** $P < 0.01$, *** $P < 0.001$.

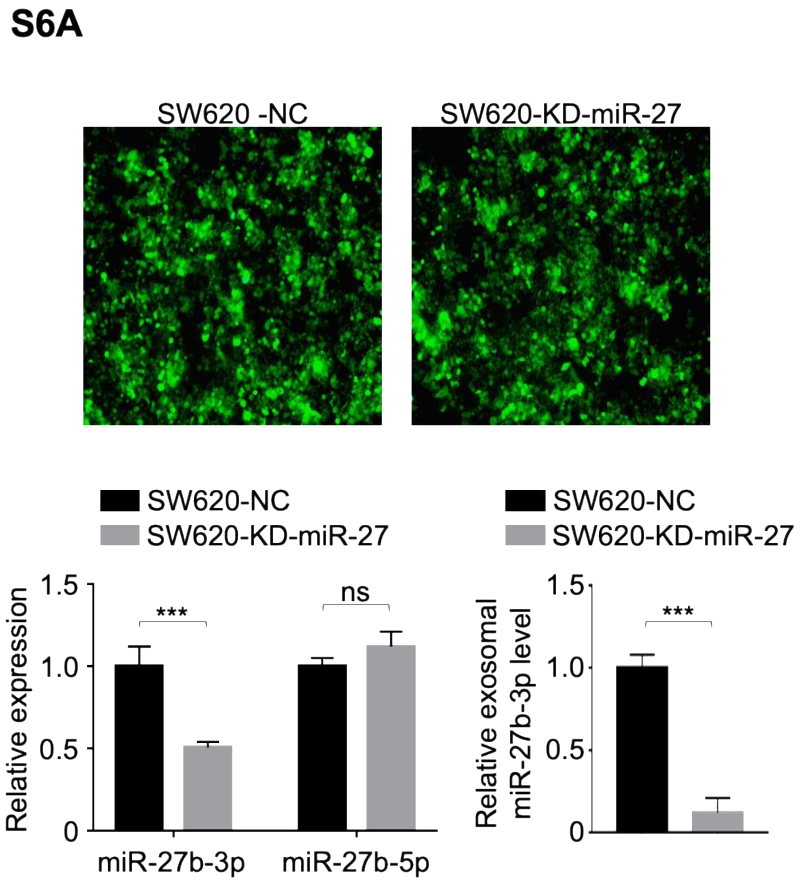

Supporting Fig.6 Colorectal cancer cell-secreted miR-27b-3p increased the permeability of endothelial monolayers in vitro

(A). Stable miR-27b-3p knock down cell lines (SW620-KD-miR-27b-3p) was established by using lentivirus, the transfection efficiency were observed with a fluorescence microscope and RT-qPCR. magnification, × 100. Error bars, SD, *** $P < 0.001$, ns, not significant.

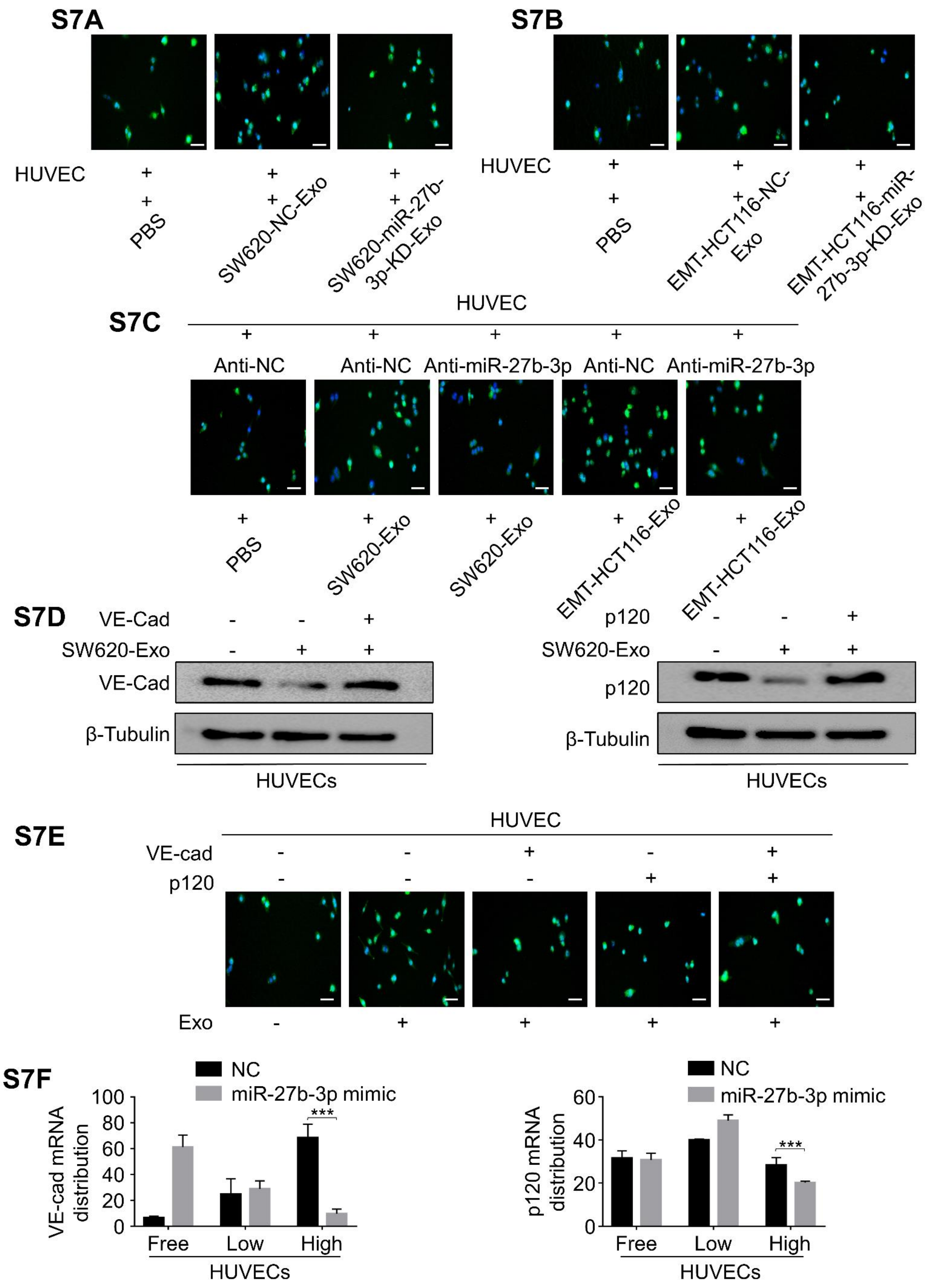

S7A
HUVEC
PBS
SW620-NC-Exo
SW620-miR-27b-3p-KD-Exo
S7B
HUVEC
PBS
EMT-HCT116-NC-Exo
EMT-HCT116-miR-27b-3p-KD-Exo
S7C
HUVEC
Anti-NC
Anti-NC
Anti-miR-27b-3p
Anti-NC
Anti-miR-27b-3p
PBS
SW620-Exo
SW620-Exo
EMT-HCT116-Exo
EMT-HCT116-Exo
S7D
VE-Cad
SW620-Exo
VE-Cad
β-Tubulin
HUVECs
p120
SW620-Exo
p120
β-Tubulin
HUVECs
S7E
HUVEC
VE-cad
p120
Exo
S7F
NC
miR-27b-3p mimic
VE-cad mRNA distribution
Free
Low
High
HUVECs
***
p120 mRNA distribution

Supporting Fig.7 Exosomal miR-27b-3p level was specific

(A)-(C). Representative images for tumor cell invasion through the HUVEC monolayer in the indicated groups. Scale bar represents 50 μm.

(D). miR-27b-3p overexpressing or miR-27b-3p/VE-Cad co-expressing HUVECs or miR-27b-3p/P120 co-expressing HUVECs. Expression of the VE-Cad, p120 proteins was determined by Western blot.

(E). Representative images for tumor cell invasion through the HUVEC monolayer in the indicated groups. Scale bar represents 50 μm.

(F). Relative distribution of VE-cad/p120 mRNA across the polysome fractions in HUVECs transfected with miR-27b-3p or NC.

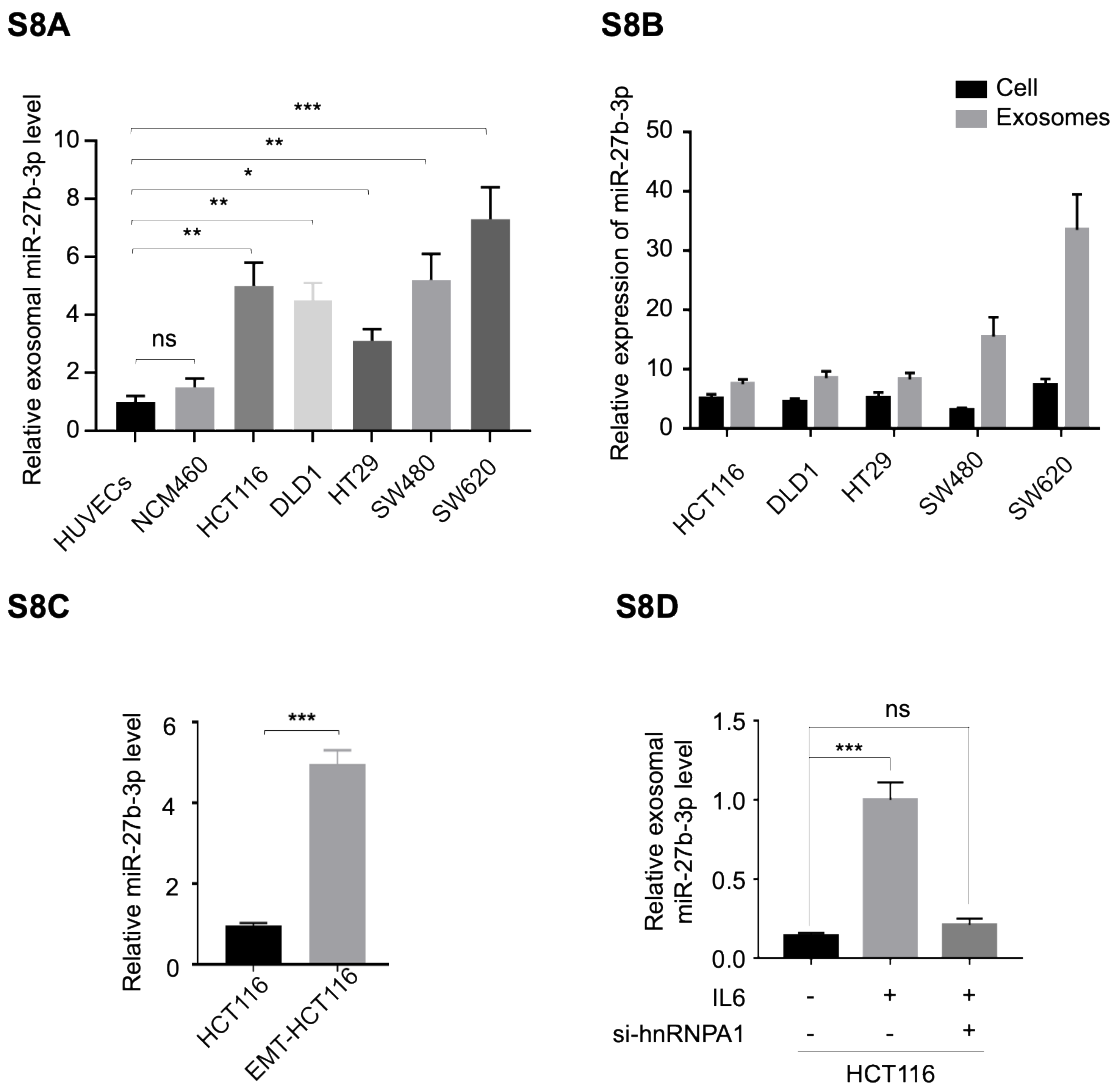

Supporting Fig.8 Exosomal miR-27b-3p level was specific

(A). The level of miR-27b-3p within HUVECs and the indicated colorectal cell lines was analyzed by qPCR. RNU6 was used as an internal control for cellular miR-27b-3p.

(B). The overall miR-27b-3p expression level in the indicated CRC cell lines and corresponding exosomes, the exosomal miR-27b-3p level did not match the primary cellular level in CRC cells.

(C). HCT116 was induced by interleukin 6 and the level of miR-27b-3p was analyzed by qPCR.

(D). qRT-PCR analysis of miR-27b-3p in CRC cells(EMT-HCT116 and SW620) and exosomes after silence hnRNPA1. Error bars, SD, * $P < 0.05$, ** $P < 0.01$, *** $P < 0.001$, ns=non-significant.

S9A

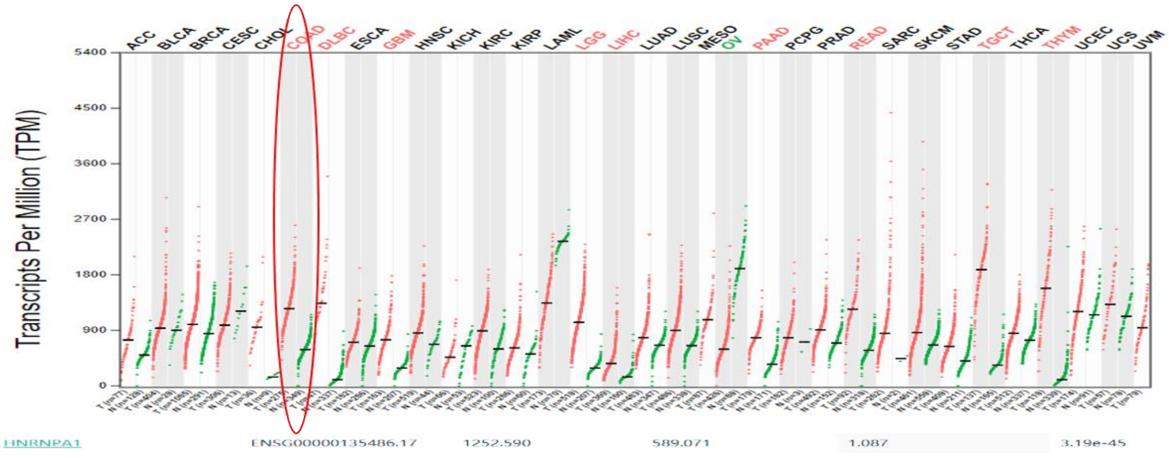

S9B

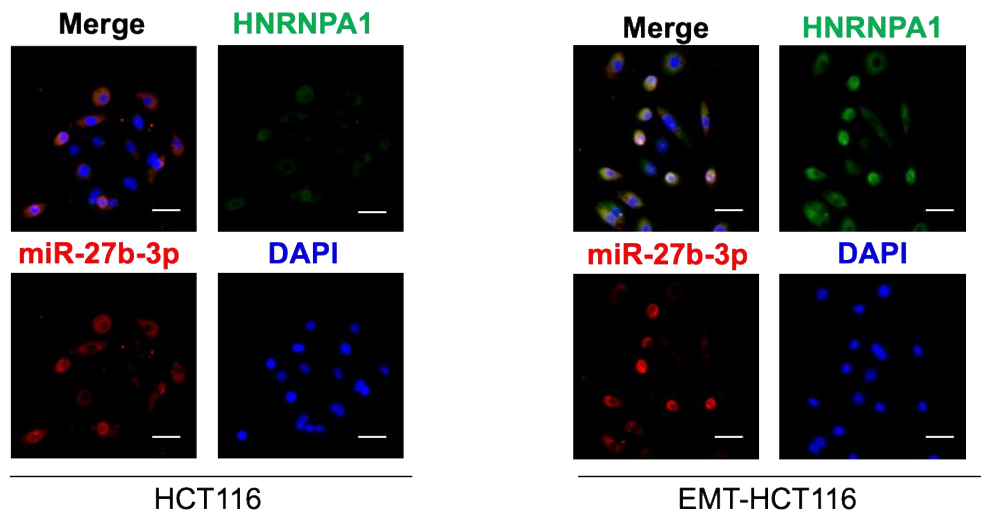

S9C

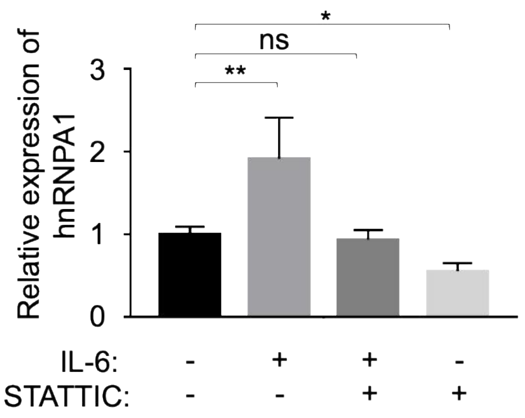

S9D

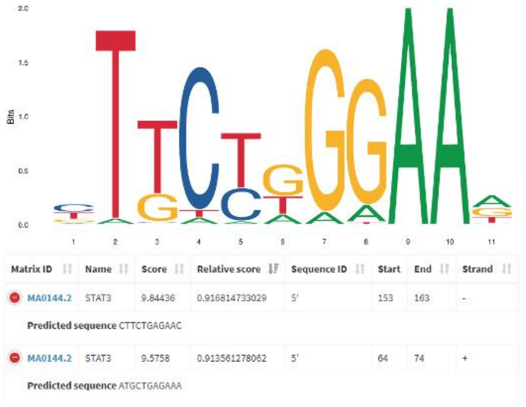

Supporting Fig.9 The packaging of miR-27b-3p into exosomes is mediated by STAT3-upregulated hnRNPA1

(A). Expression profile of hnRNPA1 in CRC tissue compared with normal tissue using Gene Expression profiling Interactive Analysis (GEPIA, http://gepia.cancer-pku.cn/index.html).

(B). Confocal microscopy image shows the co-localization of HNRNPA1 (green) and miR-27b-3p (red) in HCT116/EMT-HCT116 cells.

(C). RT-PCR was conducted to identify the expression of hnRNPA1 in the presence or absence of Stattic (15 μM). Error bars, SD, * $P < 0.05$, ** $P < 0.01$, ns=non-significant.

(D). A graphical illustration of two potential STAT3 transcriptional factor binding sites in the hnRNPA1 promoter region

S10A

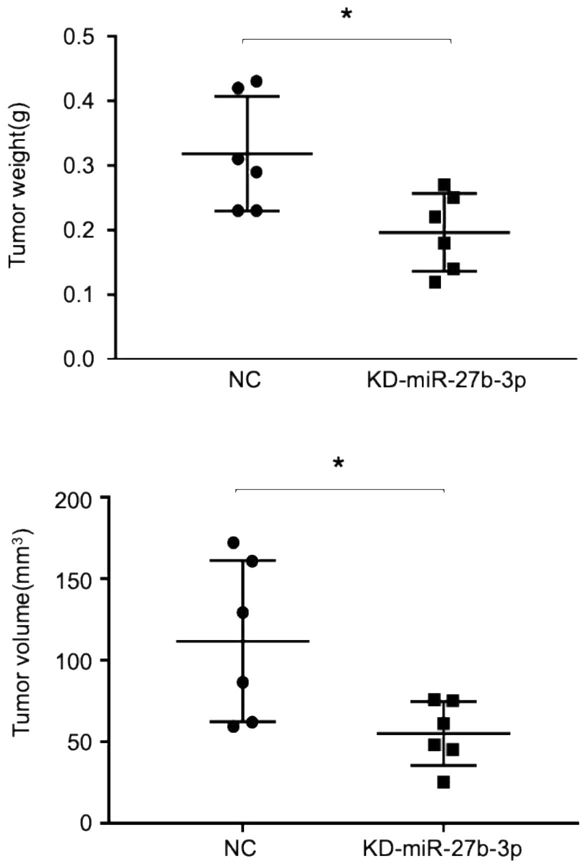

S10B

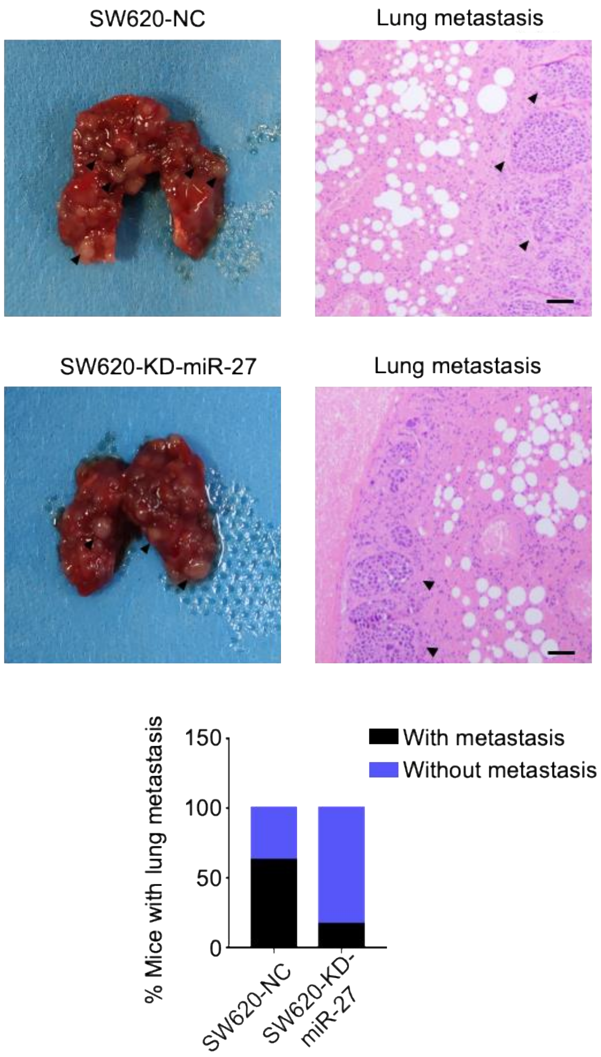

S10C

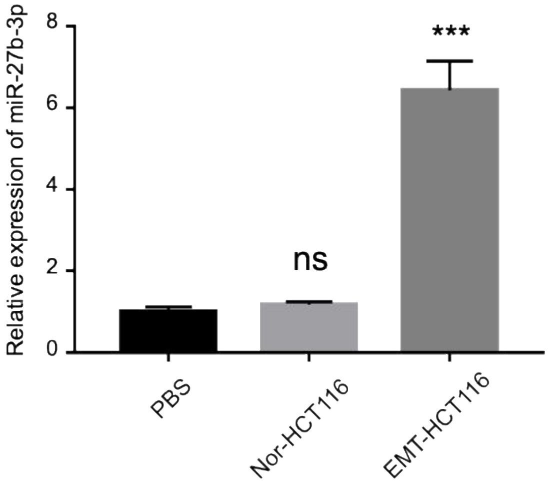

Supporting Fig.10

(A). Tumor weight and volume of SW620-NC and SW620-KD-miR-27b-3p was evaluated.

(B). Representative images of metastatic lesions in the lung from mice in the SW620-NC and SW620-KD-miR-27(n=6). Scale bar represents 100 μm. Percentage of mice with lung metastasis is indicated.

(C). qRT-PCR analysis of miR-27b-3p in HCT116 xenografts treated with intratumoral injection of indicated exosomes (n = 6 per group)

S11A

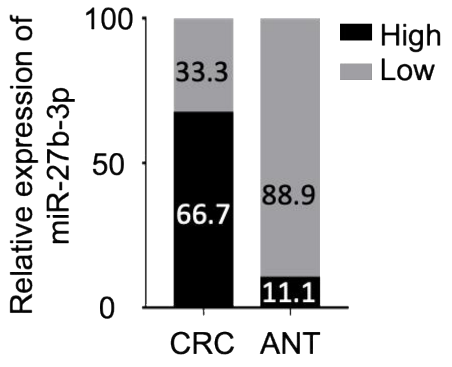

S11B

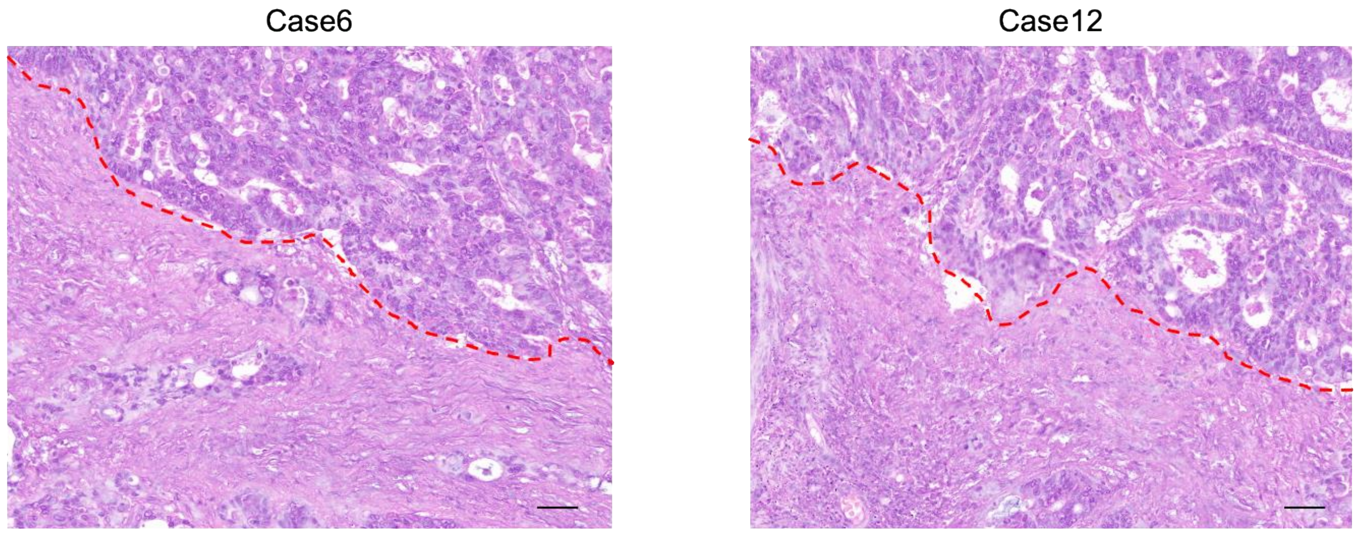

S11C

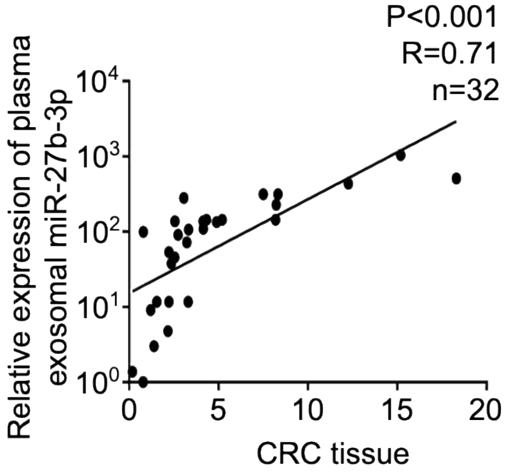

Supporting Fig11. miR-27b-3p was up-regulated in CRC cells and tissues.

(A). miR-27b-3p levels in CRC tissues were scored as described in Methods. P-value is from Chi-square (and Fisher's exact) test.

(B). Representative images of invasive front determined by HE staining from selected case 6 and case 12 CRC specimens. Scale bar represents 50 μm.

(C). Association of miR-27b-3p level between CRC tissues and circulating exosomes from CRC patients.

The sequences of the primers for qRT-PCR

Gene	Forward	Reverse
VE-cad	AAGATGCTGGCTGAGCTGTACG	GATCCAGGTTGCAATGAGGTTG
P120	GGGCTACCGGGCACCCAGTA	CGTCGAGGGTCCGAGGGTGT
β-actin	GTCATTCCAAATATGAGATGCGT	GCTATCACCTCCCCTGTGTG
E-cadherin	CACTGGGCTGGACCGAGAGAGTT	ACGCTGGGGTATTGGGGGCA
Vimentin	TTCCAAACTTTTCCTCCCTGAACC	TCAAGGTCATCGTGATGCTGAG
ZEB1	GATGACCTGCCAACAGACCA	CCCCAGGATTTCTTGCCCTT
STAT3	ACCAGCAGTATAGCCGCTTC	GCCACAATCCGGGCAATCT
hnRNPA1	GCTTTGCCTTTGTAACCTTTGACG	AGCCATCTCTTGCTTTGACAGGG
ALIX	ATCGCTGCTAAACATTACCAGTT	AGGGTCCCAACAGTATCTGGA
HRS	AGTGGCTGTCGGGTATTCATC	CCGTCCATATCCCTTGAAGAATC
GFP	AGAACGGCATCAAGGTGAAC	TGCTCAGGTAGTGGTTGTCG
miR-27b-3p	GCGCGTTCACAGTGGCTAAG	AGTGCAGGGTCCGAGGTATT
pre-miR-27b-3p	GGAAACAAAAGAAGCCACCAG	CACCCAAGCTGACTGTGCAG
CHIP1	GGGCAGGTCCTACTTTTTGC	ACCCATACTGAGCAACTAACTGC
CHIP2	TCTTCCAGAAGAGTCAGGAACC	GGTAAGTGCACAGTCTGTGTGC
CHIP-NC	ACAGCATCCACCTTTAGCTTGT	CACAGTAAGAGGCGGAGACACT
U6	CTCGCTTCGGCAGCACA	AACGCTTCACGAATTTGCGT
18s	CGGACAGGATTGACAGATTGATAGC	TGCCAGAGTCTCGTTCGTTATCG

The sequences of the siRNA

HNRNPA1: GCUGUUCUGCAGCUCAAAUTT AUUUGAGCUGCAGAACAGCTT

STAT3:GCAACAGAUUGCCUGCAUUTT AAUGCAGGCAAUCUGUUGCTT

miR-27b-3p inhibitor: GCAGAACTTAGCCACTGTGAA

Supplemental Experimental Procedures

Cell clone formation assay

Cells were transfected with miR-27b-3p mimic or miR mimic NC, miR-27b-3p inhibitor or miR inhibitor NC. 24 hours later, transfected cells were washed, trypsinized, counted, replated at a density of 500 cells/6 cm dish. 10 days later, colonies resulting from the surviving cells were fixed with 4% paraformaldehyde, stained with 0.1% crystal violet and counted. Colonies containing at least 50 cells were enumerated. Each assay was conducted in triplicates.

Wound healing assay

For wound-healing assays, Cells grow to 90-100% confluence 6-well plate, the wound is scratched by plastic Pipette tips. There maining cells were washed three times in PBS to remove cellular debris and promote at 37 ° C with serum-free medium. Migrating cells at the wound front were photographed after 24 h. All experiments were performed in triplicate. We calculated the widths of the wound by ImageJ software (http://rsbweb.nih.gov/ij/), measured and the percentage of wound healing by the formula: 100%− (Width after 24 hours / width at the beginning) × 100%.

Transendothelial invasion assay

Cell invasion assay using 24 wells Transwell (pore size 8 μm; Corning) was pre-coated Matrigel (Falcon 354480; BD Biosciences). A total of 1 × 105 cells were suspended in Add 500 μl RPMI 1640 with 1% FBS and add to Upper chamber, while 750μl RPMI 1640 contains 10% Place fetal bovine serum on the bottom. After 48 hours, cells that have migrated through the membrane and adhered The lower surface of the membrane was fixed with 4% paraformaldehyde and stained with 0.5% Crystal purple. Count and take pictures under 5 optical microscope fields (× 100 magnification). Every experiment is Repeat three times. Transendothelial invasion test was functioned to detect GFP-expressing Colorectal cancer cells that were invaded by HUVEC monolayer with or without exosome treatment.

RNA isolation and quantitative real-time PCR (qRT-PCR)

RNA was extracted using Trizol reagent (Invitrogen, USA) according to the manufacturer's instructions. miRNA quantification: Bulge-loop™ miRNA qRT-PCR Primer Sets (one RT primer and a pair of qPCR primers for each set) specific for miR-27b-3p are designed by RiboBio (Guangzhou, China). Relative expressions were calculated and normalized to endogenous GAPDH and U6. Reverse transcription of 1 μg of total RNA into cDNA using PrimeScript ™ RT kit (Toyobo, Osaka). cDNA product for subsequent qRT-PCR. Using SYBR-Green PCR Master Mix (Takara,Osaka). The quantitative real-time PCR reactions were run on a BioRad IQ5 Real time PCR machine (BioRad, USA). Relative expression was calculated using the 2-ΔΔCt method.

Western blot

RIPA buffer including a protease inhibitor cocktail (Thermo Scientific, USA) was used to lyse cells after ingestion of exosomes and the supernatant were determined using the BCA protein assay reagent kit (Aspen). The supernatants containing total protein were then mixed with 4 × SDS loading buffer and RIPA cell lysis reagent and then heated at 100°C for 10 min. Separate and transfer proteins by SDS-PAGE gel. To the PVDF membrane (Millibo, USA). After blocking with 5% skim milk, incubate at 4 ° C in primary antibody overnight. Antibodies: anti-E-cadherin (1: 1000; Cell Signaling, USA), anti-Vimentin (1: 1000; Proteintech, USA), anti-GAPDH (1: 5000; Santa Cruz, CA), anti- After incubating with ZO1 (1: 1000; Abcam, USA), anti-VE-cadherin (1: 1000; Abcam, USA), anti-Occludin(1: 1000; Proteintech, USA) ,anti-STAT3 (1: 1000; Cell Signaling, USA), anti- hnRNPA1 (1: 1000; Cell Signaling, USA) . The membranes were washed for 1 hour and placed in the HRP-conjugated secondary antibodies and incubate at room temperature for 1 hour. Bio-Rad ChemiDoc XRS was used to detect protein + system. Density analysis using Bio-Rad Image Lab software.

Luciferase assay

In briefly, HEK-293T were co-transfected with wild or mutant luciferase reporter plasmid and miR-27b-3p mimics (miR-Ribo TM negative control) using Lipofectamine 2000 (Invitrogen). 48 hours after co-transfection, the luciferase activity was determined with the Dual-Luciferase Reporter Assay System (Promega, USA). For the miRNA promoter assay, a luciferase reporter plasmid containing the full-length miR-27b-3p promoter (from -2000 to 1) was constructed and inserted into tumor cells with or without the con-transfection of STAT3. The luciferase activity was determined after 48 hours. Furthermore, miR-27b-3p promoter (-2000 to 1) and separately truncation (171-177, 171-177) were constructed as described before and inserted into pGL3-vector (Promega, USA). To mutate the potential binding sites of VEcad /p120 in miR-27b-3p promoter, the QuikChange II Site-Directed Mutagenesis Kit (Stratagene, USA) was used and inserted into pGL3-vector. Every reaction was conducted in triplicate.

Patients and clinical specimens

Human CRC tissue samples and adjacent tissues were obtained from patients who underwent total colorectal resection at

Zhong nan Hospital of Wuhan University (Wuhan, China). All included patients were diagnosed as adenocarcinoma of colorectal by pathology and 5 ml peripheral blood samples were collected in EDTA-containing tubes at the time of hospitalization to detect circulating tumor cells (CTCs). Moreover, patients underwent neoadjuvant chemotherapy or radiotherapy were excluded. All samples were collected from patients with informed consent. This study was performed with the approval of the Research Ethics Committee of Wuhan University (Wuhan, Hubei, PR China).

Cell culture and reagents

Normal intestinal epithelium cell line NCM460 and CRC cell lines (DLD-1, SW480, SW620, HCT116, and LOVO) were purchased from the Chinese Academy of Sciences in Shanghai. Human normal colon epithelial cell lines NCM460 and CRC cell lines were cultured in a 37 ° C incubator with 5% CO2 using RPMI 1640 medium (Gibco, USA) containing 10% fetal bovine serum (FBS). Transformed human embryonic kidney cells (HEK293T) use Dulbecco's modified Eagle's medium (DMEM; Life Technologies) supplemented with 10% fetal bovine serum. Human umbilical vein endothelial cells (HUVEC) were obtained from the American Type Culture Collection (ATCC). Cells were cultured in M199 medium (Invitrogen, Shanghai, China) containing 10% fetal bovine serum (FBS) (Gibco, USA) (56 °C, 30 min) fetal calf serum, streptomycin (100 U/mL), and penicillin (100 U/mL), and maintained in a humidified atmosphere of 5% CO2 at 37 °C. Recombinant human IL6 (R & D Systems) was dissolved in PBS containing 0.1% BSA at a final concentration of 50 ng / ml. Anti-human neutralizing IL-6 antibody purchased from Med Chem Express in China.

Exosomes isolation, characterization and treatment

CRC cells were cultured in RPMI-1640 medium supplemented with 10% fetal bovine serum. FBS was depleted of exosomes by ultracentrifugation using Beckman Optima L-100XP (Beckman Coulter, USA) at 110,000×g at 4 °C overnight. Cell culture medium were collected after 72h, and centrifuged at 2000g for 20min, 2,5000g for 40min at 4°C. The supernatants were passed through a 0.22 um filter and centrifuged at 110,000×g for 90min at 4°C. The amount of harvested exosomes was quantified by the BCA Protein Assay kit (Millipore, Billerica, MA, USA). Exosomes were labelled with the green-

fluorescing, lipophilic dye PKH67 according to the manufacturer's recommendations (Sigma, St. Louis, MO, USA). Briefly, exosomes were resuspended in 1 mL Diluent C mixed with 4 μLPKH67 and then co-incubated for 5 mins at room temperature. 2 ml 0.5% BSA/PBS was added to bind excess PKH67. Furthermore, the labeled exosomes were washed with PBS at 100,000 g for 1 hour, and the collecting exosomes were applied for uptake experiment.. For cell treatment, total 2 μg of exosomes were added into 2 × 105 recipient cells, incubated for 72 h.

Animal experiment

All procedures for animal experiments were carried out according to the guidelines Hubei Key Laboratory of Tumor Biological Behaviors and approved by the Institutional Animal Care and Ethical Committee in Zhong nan Hospital of Wuhan University.

For orthotropic metastasis assay, 5 × 106 growing CRC cells were subcutaneously injected on the right flank region of 4-6 weeks old female BALB/c nude mice. Once the xenografts established (within 14 days), the xenograft tumor were dissect under asepsis, and separated the fibrotic tissue from tumor, excised and minced tumor tissue into 1 mm3 pieces. Under anesthetization, both the cecum and ascending colon of the nude mice were exteriorized and the 1 mm3 pieces of xenograft were implanted sub-serosally. After suture, the bowel was returned into abdomen. For in vivo permeability assay, rhodamine–dextran(100mg/kg) was injected into the mice via tail vein. To detect the circulating tumor cells and exosomal miR-27b-3p in the blood of tumor-bearing nude mice, 1ml blood was collected in tubes with EDTA by cardiac puncture and centrifugation to isolate cells and plasma. The cells were resuspended by PBS for further CTC detection. Exosomes from plasma were further isolated by using exoRNeasy Serum/Plasma MaxiKits (QIAGEN, Germany) with the manufacturer's protocol. The RNA was extracted from exosome by using Trizol reagent and qRT-PCR were applied to quantify the miR-27b-3p level in exosome. The paraffin-embedded lungs and the livers were serial sectioned and stained with hematoxylin-eosin (HE), under microscope, metastatic nodules were screened to evaluate metastasis.

Tube formation assay, angiogenesis assay, and endothelial permeability assay.

For tube formation analysis, Matrigel was placed in a German ibidi plate (approximately 10 microliters per well) and incubated at 37 ° C for 30 minutes to polymerize Matrigel. Treated HUVECs were seeded into Matrigel-coated wells. The ibidi plates were then incubated at 37 ° C. in a humidified atmosphere of 5% CO 2. Tube formation was observed with a microscope at 12 hours. The angiogenic capacity was determined by measuring the number of tubes. The experiment was repeated 3 times. For 3D Vascular Sprouting Assays, treated HUVEC cells were suspended in M199 medium containing 0.25% (w / v) methylcellulose and seeded in non-adhesive round bottom 96-well plates (Greiner, Frickenhausen, Germany) in. All suspended cells were concentrated on the non-adherent bottom surface to form a single spheroid (in vitro angiogenesis: 500 cells per spheroid). All suspended cells aggregate to form a single spheroid (in vitro angiogenesis: 500 cells per spheroid). The spheroids were cultured for 24 hours and used for budding experiments. Matrigel Matrigel was placed in a German ibidi plate (approximately 10 microliters per well) and incubated at 37 ° C for 30 minutes to polymerize Matrigel. Individual HUVEC spheroids were individually seeded into matrigel-coated wells. The ibidi plates were then incubated at 37 ° C. in a humidified atmosphere of 5% CO 2. After 5 days, the vascular structure was imaged. Count at least 50 ellipsoids in each experiment and repeat the experiment 3 times. For Permeability assay, A single layer of treated HUVEC was grown on a 0.3 cm2 polyethylene terephthalate ultrafilter (pore size 0.4 μm; BD Biosciences; Franklin Lakes, NJ). EVOM2 voltmeter 17 (World Precision Instruments; Sarasota, FL) was used to calculate the unit area resistance. The permeability of the treated HUVEC monolayer membrane (average pore size 0.4 μm; BD Biosciences) was evaluated by the transfer of rhodamine B isothiocyanate-dextran (average MW ~ 70,000; Sigma). Specifically, rhodamine-dextran was added to the top well at a concentration of 20 mg / ml, excited at 544 nm and emitted at 590 nm using a SpectraMax microplate reader (Molecular Devices; Sunnyvale, CA), measuring 40 μl medium aliquots in a time course(1).

Transendothelial invasion assay

Cell invasion assay using 24 wells Transwell (pore size 8 μm; Corning) was pre-coated Matrigel (Falcon 354480; BD Biosciences). A total of 1 × 105 cells were suspended in Add 500 μl RPMI 1640 with 1% FBS and add to Upper chamber,

while 750μl RPMI 1640 contains 10% Place fetal bovine serum on the bottom. After 48 hours, cells that have migrated through the membrane and adhered the lower surface of the membrane was fixed with 4% paraformaldehyde and stained with 0.5% Crystal purple. Count and take pictures under 5 optical microscope fields (× 100 magnification). Every experiment is Repeat three times. Transendothelial invasion test was functioned to detect GFP-expressing Colorectal cancer cells that were invaded by HUVEC monolayer with or without exosome treatment.

CTC isolation and identification

CTC was enriched and identified by using the CTCBIOPSY® device (Wuhan YZY Medical Science and Technology Co., Ltd., Wuhan, China) as previously described by our group(2). In brief, for patients' blood sample, 2.5 ml blood was diluted into 8 ml volumes with normal saline containing 0.2% paraformaldehyde, after fixed at room temperature for 10 min, transferred to ISET tubes with 8-μm diameter aperture membrane. With a positive pressure in the range from 12 to 20mmHg, the candidate CTCs were adhered to the aperture membrane and stained with three-color immunofluorescence staining. Immunofluorescence protocol was described before.

Transfection of miR-27 mimic, inhibitor, and siRNA of the target gene

Hsa-miR-27 mimic and mimic negative control (NC), hsa-miR-27 inhibitor, and inhibitor negative control (NC) were purchased from RiboBio (Guangzhou, China).

VE-Cad / P120 siRNAs were obtained from GenePharma (Shanghai, China). Cells were cultured in complete medium at least 24h before transfection. Cells were washed with phosphate-buffered saline (PBS, pH 7.4) before transient transfection. Transfections were performed by Lipofectamine 2000 (Invitrogen ,USA) according to the manufacturer's protocol with RNA oligonucleotides at a final concentration of 50 nM.

RNA immunoprecipitation (RIP) assay

RIP assays were performed using a RIP protein immunoprecipitation kit (Millibo, Massachusetts, USA). Briefly, the collected cells (the number of cells in each group is about 5 × 107) are lysed in ice-cold polysome lysis buffer. Supplemented

with protease inhibitors, RNase inhibitors, and 1 mM PMSF in the mixed solution. The lysate was placed in an ultra-highspeed centrifuge at 4 ° and centrifuged at 14,000 × g for 15 minutes. 50 μL of lysate was saved as input. The protein extract (1 mg) was induced with 3 μg of rabbit anti-hnRNPA1 antibody (Cell Signaling Technology, USA) or rabbit IgG (Proteintech, USA) overnight at 4 ° C with end- Over-end rotation. Approximately 30 μL of A / G protein magnetic beads was then added and promoted at 4 ° C for 4 h. The magnetic beads were then washed five times to elute unbound substances. After digesting protein with proteinase K, the isolated miRNA was reverse transcribed and then analyzed by real-time PCR.

Chromatin immunoprecipitation (ChIP) assay

ChIP assays were performed with Cell Signaling SimpleChIP®Enzymatic Chromatin IP Kit (#9003, USA) according to the manufacturer's instructions. In brief, HCT116 cells were fixed by using 1% formaldehyde and incubate 10 min at room temperature. The fixed product was collected and digested with Micrococcal Nuclease. Antibodies against STAT3(1:50, Cell Signaling, USA) and rabbit IgG (1:500, Cell Signaling, USA) were added into lysed for immunoprecipitation. After elution and reversal of cross-links, qRT-PCR were applied to quantify the precipitated DNA, primers for CHIP1, CHIP2 and negative control were listed in Supplementary Table.

Fluorescence in situ hybridization and in situ hybridization

Paraffin- embedded tissue blocks were serially sectioned into 4-μm thickness. FISH was performed in tissue sections by using in situ hybridization (ISH) kit and fluorescence in situ hybridization (FISH) kit (Bosterbio, USA) separately according to the manufacturer's protocol. The miR-27b-3p detection probe was synthesized by Bosterbio.

Immunofluorescence

Tissue sections (4 μm thick) or cells grown on cover slips were fixed with formalin (containing 0.1% DEPC) for 20 min. Cells were washed with PBS. After permeabilization with 0.4% pepsin for 5-120s, blocking buffer (3% BSA) was added. Samples were then incubated with primary antibodies, followed stain with FITC-conjugated anti-mouse secondary antibody (Abcam, USA, 1:1000) or Cy3- conjugated anti-rabbit secondary antibody (Abcam, USA, 1:1000). Nuclei were stained

with DAPI. Section and cell were observed and images were acquired using an Olympus BX53 fluorescence microscope. Protein expression was quantified using a visual grading system based on the extent of staining (the propotion of positive cells on a scale of 0–4: 0, none; 1, 1%–25%; 2, 26%–50%; 3, 51%–75%; 4, >75%) and the immunostaining intensity (graded on a scale of 0–3: 0, no staining; 1, weak staining; 2, moderate staining; 3, strong staining), the product is the expression intensity

Plasmid constructs

The genomic sequences of human VE-cadherin and p120-3'UTR were amplified by PCR and cloned into the XhoI/NotI site of pmiR-RB-Report™ luciferase reporter vector (RiboBio, Guangzhou, China), their corresponding mutant (Mut) 3'-UTR sequences were generated by site directed PCR-mutagenesis and inserted into the same sites of pmiR-RB-Report™ luciferase reporter vector. All genomic products were confirmed by sequencing. Primers for this experiment were listed in Supplementary Table.

Statistical analysis

All results were carried out more than three independent experiments, and finally confirmed. Unless otherwise specified, all experimental data provided were expressed by means. All statistical analyses used GraphPad Prism software (version 6.0, GraphPad software, United States) and SPSS statistical software (version 22.0, IBM SPSS, United States). Two-tailed Student's tests were used to compare the means Quantitative data between groups, or when more than two groups were compared by one-way analysis of variance. Values of $P < 0.05$ are considered statistically significant. The Kaplan–Meier method was used to calculate the overall survival rate, and the difference was calculated and analyzed by the log-rank test.

Reference

1. Zhou W, Fong MY, Min Y, Somlo G, Liu L, Palomares MR, et al. Cancer-secreted miR-105 destroys vascular endothelial barriers to promote metastasis. Cancer Cell. 2014;25(4):501-15.

2. Wang Y, Fang W, Huang Y, Hu F, Ying Q, Yang W, et al. Reduction of selenium-binding protein 1 sensitizes cancer cells to selenite via

elevating extracellular glutathione: a novel mechanism of cancer-specific cytotoxicity of selenite. Free Radic Biol Med. 2015;79:186-96.
